# Seascape genetics and biophysical connectivity modelling support conservation of the seagrass *Zostera marina* in the Skagerrak–Kattegat region of the eastern North Sea

**DOI:** 10.1111/eva.12589

**Published:** 2018-01-26

**Authors:** Marlene Jahnke, Per R. Jonsson, Per‐Olav Moksnes, Lars‐Ove Loo, Martin Nilsson Jacobi, Jeanine L. Olsen

**Affiliations:** ^1^ Department of Marine Sciences – Tjärnö University of Gothenburg Strömstad Sweden; ^2^ Groningen Institute for Evolutionary Life Sciences Section: Ecology and Evolutionary Genomics in Nature (GREEN) University of Groningen Groningen The Netherlands; ^3^ Department of Marine Science University of Gothenburg Gothenburg Sweden; ^4^ Complex Systems Group Department of Energy and Environment Chalmers University of Technology Gothenburg Sweden

**Keywords:** barrier analysis, conservation, directional dispersal, isolation by oceanography, Lagrangian particles, seascape genetics

## Abstract

Maintaining and enabling evolutionary processes within meta‐populations are critical to resistance, resilience and adaptive potential. Knowledge about which populations act as sources or sinks, and the direction of gene flow, can help to focus conservation efforts more effectively and forecast how populations might respond to future anthropogenic and environmental pressures. As a foundation species and habitat provider, *Zostera marina* (eelgrass) is of critical importance to ecosystem functions including fisheries. Here, we estimate connectivity of *Z. marina* in the Skagerrak–Kattegat region of the North Sea based on genetic and biophysical modelling. Genetic diversity, population structure and migration were analysed at 23 locations using 20 microsatellite loci and a suite of analytical approaches. Oceanographic connectivity was analysed using Lagrangian dispersal simulations based on contemporary and historical distribution data dating back to the late 19th century. Population clusters, barriers and networks of connectivity were found to be very similar based on either genetic or oceanographic analyses. A single‐generation model of dispersal was not realistic, whereas multigeneration models that integrate stepping‐stone dispersal and extant and historic distribution data were able to capture and model genetic connectivity patterns well. Passive rafting of flowering shoots along oceanographic currents is the main driver of gene flow at this spatial–temporal scale, and extant genetic connectivity strongly reflects the “ghost of dispersal past“ sensu Benzie, 1999. The identification of distinct clusters, connectivity hotspots and areas where connectivity has become limited over the last century is critical information for spatial management, conservation and restoration of eelgrass.

## 1 INTRODUCTION

Eelgrass (*Zostera marina* L.) is one of the most widely distributed species of seagrass in the northern hemisphere and the dominating species of the temperate North Atlantic (Short, Carruthers, Dennison, & Waycott, 2007). It is a benthic foundation species within shallow coastal areas where it provides habitat and numerous ecosystem services, such as stabilization of the coastline and improved water quality, increased fish production and uptake of carbon and nitrogen (Cole & Moksnes, 2016; Orth et al., 2006). Large‐scale losses of eelgrass have occurred worldwide (Waycott et al., 2009), including northern Europe (Boström et al., 2014), causing significant decreases of ecosystem services. For example, along the Swedish Skagerrak coast, a reduction of 120 km^2^ has resulted in large losses of cod catches, and release of sequestered carbon and nitrogen, to an estimated total cost of more than 600 million US$ (Cole & Moksnes, 2016). In response, *Z. marina* has been classified as a ‐threatened and declining habitat‐ in the North East Atlantic and the Baltic Sea under regional marine conventions (HELCOM 2010; OSPAR 2017) and is also indirectly protected under several EU directives including the Habitats Directive (92/43/EEC) and its Natura 2000 network.

The largest known areal distribution of *Z. marina* in Europe is found in the Skagerrak–Kattegat–Belt Sea region in the eastern part of the North Sea (Boström et al., 2014). As in most parts of the North Atlantic, a dramatic loss of *Z. marina* occurred in the area in the 1930s, as a result of the wasting disease (Rasmussen, 1977). Eelgrass recovered in most areas in the 1960–1980s, but never obtained its historic distribution (Boström et al., 2014). In the following decades, *Z. marina* distribution in Denmark decreased again, probably as a result of nutrient pollution (Boström et al., 2014). It is estimated that eelgrass in Denmark today constitutes 10%–20% of its historic distribution and that the depth distribution has become more shallow by approximately 50%, resulting in a loss of most offshore populations (Boström, Baden, & Krause‐Jensen, 2003; Boström et al., 2014). Along the Swedish Skagerrak coast, over 60% of meadows have been lost since the 1980s (Baden, Gullström, Lundén, Pihl, & Rosenberg, 2003; Nyqvist, André, Gullström, Baden, & Åberg, 2009). These losses have largely been attributed to coastal eutrophication and overfishing of large predatory fish, causing a trophic cascade and an increase in ephemeral macroalgae that smother *Z. marina* (Baden, Emanuelsson, Pihl, Svensson, & Åberg, 2012; Moksnes, Gullström, Tryman, & Baden, 2008). To mitigate the ongoing loss and assist recovery of *Z. marina*, a number of measures are presently being discussed, including the establishment of new networks of marine‐protected areas (MPAs) and large‐scale restoration (Moksnes et al., 2016; SwAM 2015).

We hypothesize that the drastic decline of *Z. marina* in the Skagerrak–Kattegat region over the past 140 years has directly impacted meta‐population dynamics by creating a much reduced and more fragmented eelgrass seascape. From a population genetics perspective, this condition may have led to reduced migration, lower effective population size and loss of allelic diversity (through genetic drift), resulting in decreased evolutionary potential to adapt to changing environments (Allendorf, Luikart, & Aitken, 2013; Leimu, Mutikainen, Koricheva, & Fischer, 2006). Genetic diversity and connectivity are also crucial from an ecological perspective for growth and persistence of local populations (Baguette, Blanchet, Legrand, Stevens, & Turlure, 2013; Lagabrielle et al., 2014). Seagrass systems (and *Z. marina* in particular) have been extensively studied in a population genetics framework (reviewed in Procaccini, Olsen, & Reusch, 2007). Experimental studies focusing on genetic–diversity–ecosystem–function relationships show that high genotypic richness leads to greater resilience and resistance (Hughes & Stachowicz, 2004; Reusch, Ehlers, Hämmerli, & Worm, 2005) and higher productivity (Hughes, Inouye, Johnson, Underwood, & Vellend, 2008), and high allelic richness leads to increased restoration success and ecosystem services (Reynolds, McGlathery, & Waycott, 2012). Population genetics have also been used to understand how dispersal and gene flow affect temporal–spatial population structure of seagrasses (Hernawan et al., 2016; Jahnke et al., 2016; Sinclair et al., 2016; Talbot et al., 2016), which in *Z. marina* is driven by dispersal via pollen or negatively buoyant seeds in the range of metres (McMahon et al., 2014; Orth, Luckenbach, & Moore, 1994; Reusch, Boström, Stam, & Olsen, 1999; Reusch, Stam, & Olsen, 1999), and long‐distance dispersal over 10s – 100s km via surface‐floating flowering shoots (Harwell & Orth, 2002; Hosokawa, Nakaoka, Miyoshi, & Kuwae, 2015; Källström, Nyqvist, Åberg, Bodin, & André, 2008; Kendrick et al., 2012; McMahon et al., 2014) or via grazing waterfowl and fish (Sumoski & Orth, 2012).

The Skagerrak–Kattegat region is particularly well suited to study the potential impact of large‐scale decline on connectivity, due to the availability of detailed historic data of eelgrass as well as unusually good mapping of the current distribution (Boström et al., 2014). In addition, the oceanographic features of the area are unique, with a strong outflow of surface water from the Baltic Sea into the Kattegat, creating an asymmetric circulation along the coasts with strong effects on connectivity, also creating a barrier between the Kattegat and Skagerrak (Jonsson, Corell, André, Svedäng, & Moksnes, 2016; Jonsson, Nilsson Jacobi, & Moksnes, 2016; Leppäranta & Myrberg, 2009). Biophysical dispersal models, such as Lagrangian trajectory models (Cowen & Sponaugle, 2009; Grech et al., 2016; Selkoe et al., 2010), can model such directional dispersal based on biologically realistic assumptions, for example, time of propagule release, drift duration and depth, and may be superimposed on layers of habitat preference.

Here, we aim to establish a dynamic model of seascape population structure and connectivity for *Z. marina* meadows in the Skagerrak–Kattegat region. Our assessment includes a temporal comparison based on oceanographic dispersal modelling of extant and historical distribution data of *Z. marina* for the region, and we investigate the hypothesis that the large observed decline has resulted in decreased connectivity and lower genetic diversity. In addition, we examine how oceanographic and genetic barriers fit with present administrative borders such as countries and sea basins. We combine and cross‐validate genetic and hydrodynamic modelling approaches in order to infer the importance of dispersal in shaping population structure and to compare trade‐offs and synergies offered by the integration of the two approaches as applied to management and mitigation.

## 2 MATERIAL AND METHODS

### 2.1 Study area and sampling

**Figure 1:**
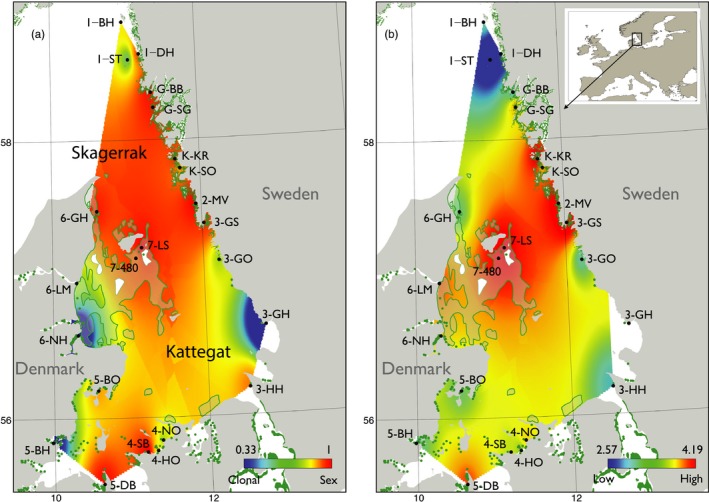
Map of sampling sites for *Zostera marina* in the Skagerrak–Kattegat region of the North Sea (Table 1). Green dots indicate the extant mapped distribution of *Z. marina*; the area enclosed by the solid green line in western Kattegat shows the estimated historic distribution of *Z. marina*. The background heat map in (a) shows an interpolation of genotypic/clonal diversity; (b) allelic richness standardized for 21 genotypes (A_21_) generated with the genetic diversity plugin (Vandergast, Perry, Lugo, & Hathaway, 2011) in ArcMap 10.3 (Desktop, 2014) and QGIS 2.18 (Quantum GIS Development Team 2013)

The study covers the eastern Skagerrak, Kattegat and Belt seas along the eastern part of the North Sea (54–59°N and 8–13°E) with a total area of 77,000 km^2^ (Figure 1). For simplicity, we refer to the assessed area as Skagerrak–Kattegat. Sampling of *Z. marina* was guided by a previous oceanographic barrier analysis of the region (Moksnes, Jonsson, & Nilsson Jacobi, 2015; Nilsson Jacobi, André, Döös, & Jonsson, 2012) in which seven oceanographic clusters were identified (site names in Figure 1 follow the seven clusters). At least three sites were sampled from each of the seven oceanographic clusters (from here on we, will refer to these clusters as sampling areas) to ensure sampling within and across potential barriers to dispersal. At each site, 40 shoots were collected using a ‐roughly linear swim‐ (Arnaud‐Haond, Duarte, Alberto, & Serrão, 2007) by snorkelling or diving. Within sites, intersample distance was maintained at 1–1.5 m (covering a distance of 40–60 m across a meadow), a standard distance for this species and an adequate compromise to capture diversity and structure, while minimizing resampling of the same genotype (Olsen et al., 2004). Sample depths ranged from 1.2 to 5.3 m. A total of 920 sampling units were collected. Among sites, pairwise distances ranged from ~10 to 400 km among the 23 sampling sites. Similar sampling scales between sites were maintained as much as possible to best detect a slow decline in allele frequency, that is, 8% of sites have a pairwise geographic distances of up to 50 km, 21% up to 100 km, 23% up to 150 km, 22% up to 200 km, 17% up to 300 km and 8% up to 400 km. Samples were collected in July and August 2016 except for two populations from Denmark (6‐NH and 5‐BO), which were collected during a previous sampling campaign in 2004 (Table 1). One to three 2‐cm leaf pieces per shoot were selected from the clean, inner leaves near the base of the shoot and when necessary cleaned of epiphytes with a scalpel. Samples were dried and stored in silica crystals for later DNA extraction.

**Table 1:** Genetic diversity of *Zostera marina* at 23 locations in the Skagerrak–Kattegat region of the North Sea

Population	Acronym	Latitude	Longitude	*N*	*MLG*	*R*	*A*_21_ (*SD*)	*H*_O_ (*SE*)	*H*_E_ (*SE*)	*F* (*SE*)
Borholmen	1‐BH	10.99483	58.85127	40	32	.79	3.25 (0.12)	0.31 (0.05)	0.31 (0.05)	0.03 (0.04)
Dannholmen	1‐DH	11.22188	58.61912	40	36	.90	2.86 (0.09)	0.39 (0.05)	0.37 (0.05)	−0.05 (0.06)
Storön	1‐ST	11.0705	58.57873	40	28	.69	2.57 (0.06)	0.37 (0.05)	0.39 (0.05)	**0.09 (0.04)**
Bubacka	G‐BB	11.3702	58.34075	40	39	.97	3.21 (0.09)	0.38 (0.04)	0.40 (0.04)	0.03 (0.03)
Gåsö	G‐SG	11.39633	58.2315	40	38	.95	3.74 (0.17)	0.38 (0.05)	0.37 (0.05)	−**0.04 (0.02)**
S Kråkerön	K‐KR	11.669	57.856	40	37	.92	4.17 (0.16)	0.33 (0.05)	0.35 (0.05)	**0.06 (0.05)**
N St Överön	K‐SO	11.73167	57.79033	40	34	.85	3.78 (0.14)	0.45 (0.05)	0.44 (0.04)	−0.02 (0.04)
Malevik	2‐MV	11.92637	57.52893	40	40	1.00	4.15 (0.18)	0.29 (0.05)	0.36 (0.06)	**0.21 (0.07)**
Gottskär	3‐GS	12.02328	57.38913	40	37	.92	4.19 (0.12)	0.33 (0.06)	0.32 (0.05)	0.03 (0.08)
Getterö	3‐GO	12.20353	57.11842	40	30	.74	3.27 (0.09)	0.39 (0.05)	0.37 (0.05)	−**0.05 (0.04)**
Grötvik Hamn	3‐GH	12.77905	56.6415	40	14	.33	na	0.42 (0.05)	0.41 (0.05)	0.02 (0.05)
Högenäs Hamn	3‐HH	12.53337	56.19758	40	35	.87	3.19 (0.11)	0.39 (0.04)	0.43 (0.04)	**0.09 (0.03)**
N Ordrup	4‐NO	11.38543	55.8351	40	30	.74	3.66 (0.16)	0.33 (0.05)	0.33 (0.05)	0.00 (0.03)
Hamnsö	4‐HO	11.31785	55.76127	40	32	.79	3.42 (0.15)	0.36 (0.05)	0.36 (0.05)	0.02 (0.04)
Saltbäk	4‐SB	11.18587	55.75207	40	40	1.00	3.5 (0.11)	0.34 (0.05)	0.34 (0.05)	−0.01 (0.03)
Dalby Bay	5‐DB	10.6243	55.5273	40	39	.97	3.93 (0.16)	0.38 (0.05)	0.42 (0.05)	**0.06 (0.04)**
Bisholt	5‐BH	9.977233	55.82987	40	21	.51	3.3 (0)	0.38 (0.05)	0.42 (0.05)	**0.09 (0.03)**
Bogens	5‐BO	10.57	56.2	40	34	.85	3.35 (0.10)	0.41 (0.06)	0.41 (0.05)	0.01 (0.04)
Norhold	6‐NH	10.32	56.6	40	16	.38	na	0.38 (0.05)	0.36 (0.05)	0.02 (0.06)
Limfjord	6‐LM	10.31062	56.97795	40	30	.74	3.91 (0.08)	0.40 (0.05)	0.36 (0.05)	−**0.09 (0.03)**
Grholm	6‐GH	10.59772	57.49155	40	38	.95	3.34 (0.12)	0.36 (0.05)	0.37 (0.05)	**0.08 (0.07)**
Læsø	7‐LS	11.18207	57.22405	40	40	.74	4.15 (0.13)	0.35 (0.05)	0.33 (0.04)	−0.03 (0.04)
Læsø	7‐480	11.10238	57.14862	40	36	.90	4.05 (0.08)	0.40 (0.05)	0.42 (0.05)	**0.04 (0.04)**

The 920 individuals sampled in Denmark and Sweden were assessed with 20 microsatellites. Population names are followed by the acronyms, latitude and longitude, the number of sampled ramets (*N*), the number of multilocus genotypes (*MLG*), genotypic richness (*R*) as *MLG‐1/N‐1,* allelic richness standardized to 21 genotypes (*A*_21_) plus standard deviation (*SD*), not applicable (na) due to low number of *MLG*s, observed heterozygosity (*H*_O_), expected heterozygosity (*H*_E_) and the inbreeding coefficient (*F*), and standard error (*SE*). Numbers in bold indicate significant *F* values.

### 2.2 DNA extraction and microsatellite amplification

DNA was extracted from ~20 mg of silica‐gel‐dried tissue in 96‐well plates using a silica‐based Cetyl trimethylammonium bromide (CTAB) protocol (Hoarau, Coyer, Stam, & Olsen, 2007), except that samples were incubated in CTAB for 1 hr at 60°C. Twenty‐two microsatellite loci were used: the original set of eight from Reusch, Boström et al. (1999), Reusch, Stam et al. (1999) and used in numerous genetic surveys of *Z. marina*, and 14 additional loci developed from expressed sequence tags (EST) libraries (Keil, 2011; Oetjen & Reusch, 2007). Primer sequences, multiplex combinations and concentrations are provided in Tables S1 and S2. Polymerase chain reactions (PCRs) were performed in 96‐well microtiter plates using the Qiagen Kit Type‐IT^®^ in a 6.2 μl reaction volume following the manufacturer's instructions. The reaction profile consisted of 95°C for 5 min followed by 30 cycles of 95°C for 30 s, 56°C for 1 min 30 s and 72°C for 30 s, with a final extension step of 60°C for 30 min.

### 2.3 Microsatellite genotyping, removal of clones, data quality checks and discrimination power

PCR products were diluted 1:100 (apart from the “4‐plex,“ which was used undiluted), and fragment analysis was performed on an Applied Biosystems 3730 DNA Analyser with a 350 ROX internal size standard added to each well. Fragments were scored automatically using GeneMapper^®^ (Life technologies) and re‐checked by eye for each individual and locus. Samples with ambiguous or rare alleles were re‐amplified and re‐genotyped for confirmation. We succeeded in amplifying all individuals at all loci.

Because seagrasses can spread clonally via rhizome extension, a genetic individual (genet) may consist of hundreds or thousands of shoots (ramets) covering several metres. Even though a sampling distance of 1–1.5 m is generally adequate for *Z. marina* (Olsen et al., 2004), it is no guarantee that the same genet might not be sampled more than once if large clones are present. Accordingly, duplicate multilocus genotypes (*MLG*s) were identified and removed using RClone (Bailleul, Stoeckel, & Arnaud‐Haond, 2016) in R 3.3.1 (R Development Core Team 2014). Only one *MLG* for each clone was retained. The method is based on the probability that identical *MLG*s have not arisen by chance via sexual reproduction (*p*_sex_(*F*_IS_)) taking into consideration Hardy‐Weinberg equilibrium (HWE), and a threshold of 0.05.

Null alleles were tested for with MicroDrop (Wang & Rosenberg, 2012; 10,000 permutations and 100 replicates), because the method does not rely on HWE assumptions to calculate null allele frequencies. Linkage disequilibrium (LD) and HWE were evaluated for each locus and across all loci in each population with Genepop 4.2 (Raymond & Rousset, 1995; 100 batches and 1,000 iterations per batch plus Bonferroni corrections). To test for neutrality of our loci, “outlier“ analyses were performed using both Lositan (Antao, Lopes, Lopes, Beja‐Pereira, & Luikart, 2008) and BayeScan (Foll & Gaggiotti, 2008). See Figure S1 for additional information.

To analyse the statistical power of our set of microsatellites to discriminate clonal replicates, we calculated the probability of identity (PI) in GenAlEx 6.5 (Peakall & Smouse, 2012) at each site and used POWSIM 4.1 (Ryman & Palm, 2006) to evaluate the statistical power to detect population structure among sites. For POWSIM, we used the observed allele frequencies, sampling sites and *MLG*s to simulate drift to *F*_ST_ values of 0, 0.001, 0.01 and 0.1 using an effective population size (*N*_e_) of 200 and a range of generations t (0–100) with 1,000 replicates and 100,000 batches.

### 2.4 Genetic diversity and population differentiation

We calculated heterozygosity‐based estimates in GenAlEx 6.5 (Peakall & Smouse, 2012). Genotypic diversity (also called clonal diversity) was calculated based on *MLG* identification (as described above) with the formula *MLG*‐1/N‐1, where *N* is the number of ramets (Dorken & Eckert, 2001). Allelic richness standardized to the same number of *MLG*s was calculated with *standArich* (http://alberto-lab.blogspot.nl/p/code.html#!/p/code.html) in R 2.15.3.

To gain a first impression of genetic structure of all *MLG*s without a priori population genetic assumptions, we used PCA as implemented in *adegenet* 2.0.1 (Jombart, 2008) in R 3.3.2 after using the scaleGen function. PCAs were also used to investigate potential outlier loci and for visualization of the distribution of our sampling sites in a larger geographic context (see Supporting Information). Population genetic differentiation was calculated as the proportion of shared alleles among populations, *D*_ps_’ = 1 – ps, in MSA 4.05 (Dieringer & Schlötterer, 2003). We chose *D*_ps_, because it is free of equilibrium assumptions (Bowcock et al., 1994). We also calculated several standard variance‐based measures of population differentiation (Tables S5–S7).

Spatial genetic structure was analysed in a Bayesian framework using two methods: Structure 2.3.3 (Pritchard, Stephens, & Donnelly, 2000) and TESS 2.3 (Chen, Durand, Forbes, & François, 2007). Structure cannot always identify clusters accurately when geographic sampling is discrete along clines and/or when isolation‐by‐distance (IBD) patterns or autocorrelations dominate the data (Chen et al., 2007). TESS addresses these issues using a spatially continuous prior based on the geographic coordinates of each individual. Therefore, our main analyses rely on TESS results, while Structure was carried out as an additional support. As we had geographic information only at the meadow level, we used TESS to calculate slightly adapted geographic coordinates for each individual. TESS was run using the conditional autoregressive Gaussian (CAR) admixture model, which assumes spatial autocorrelation of genetic differentiation, using the default value of 0.6 for the strength of the autocorrelation. We first ran a test with default settings for *K*_max_ = 2–25 and then repeated TESS for a range of likely *K*_max_ (2–7) with a burn‐in of 10,000 sweeps followed by 25,000 sweeps, with 100 independent runs conducted for each *K*_max_. The independent runs were averaged and compared to assess convergence. The average deviance information criterion (DIC) for each value of *K*_max_ was used to evaluate the most likely number of genetic clusters by determining *K*_max_ at which a higher number of parameters did not improve the model significantly. We used *pophelper* (Francis, 2016) with CLUMPP 1.1.2 (Jakobsson & Rosenberg, 2007) in R 3.2.2 for postprocessing of TESS outputs and visualization of clusters. To display ancestry coefficients (proportion of each individual belonging to each cluster) geographically, we used the script provided at http://membres-timc.imag.fr/Olivier.Francois/TESS_Plot.html.

### 2.5 Directional migration

We use the term “migration“ in the population genetic sense, which includes both successful movement and contribution to the local gene pool (Lowe & Allendorf, 2010). In contrast, we use “dispersal“ when discussing movement based on oceanographic modelling to reflect a passive process of transport influenced by currents but that does not necessarily result in any contribution to a local gene pool. Directional migration rates based on the microsatellite data were estimated using two different methods: DivMigrate‐online (https://popgen.shinyapps.io/divMigrate-online/) and GENECLASS2 (Piry et al., 2004). DivMigrate is an indirect approach that extends genetic differentiation to include a directional measurement by identifying migrants based on the geometric means of the allele frequencies in each population. Directional migration rates are then inferred from allele frequencies and genetic differentiation in pairwise comparisons of *G*_ST_ (Sundqvist, Keenan, Zackrisson, Prodohl, & Kleinhans, 2016). In contrast, GENECLASS2 (Piry et al., 2004) is a direct approach that uses an assignment test to identify first‐generation migrants. The advantage of assignment tests is that they do not rely on HWE. Their main disadvantage is that generally few first‐generation migrants are identified in benthic species (see for instance Lukoschek, Riginos, & van Oppen, 2016; Jahnke et al., 2017). Although we had originally planned to use BayesAss (Rannala 2007) and Migrate (Beerli & Felsenstein, 2001), we were unable to do so because of problems with convergence and repeatability of results as also reported in other studies (Epps & Keyghobadi, 2015; Meirmans, 2014).

### 2.6 Mapping of suitable habitat

Data for present‐day distribution of *Z. marina* were based on national inventories in Norway, Sweden and Denmark, and were obtained in geographic information system (GIS) format from the Norwegian Environment Agency, the Swedish County Administrative Board of Västra Götaland and the Danish Nature Agency. Along the Swedish Skagerrak coast, distribution was based on satellite image analyses (Envall & Lawett, 2016), whereas the distribution in other areas was based on national field surveys and monitoring sites. In the oceanographic modelling, all grid cells that intersected with eelgrass locations were used as sources in the particle tracking simulation and subsequent construction of the connectivity matrices.

In addition to mapping the present distribution of *Z. marina*, we also explored the effect of including the known historic distribution on multigeneration connectivity. These data were obtained from a recent analysis of historic records of *Z. marina* presence in the Kattegat collected around 1900 (Petersen, 1893; Rosenvinge, 1909). The historic collection sites were revisited in 2015–2016 to confirm historic depth data and map the present distribution. A total of 1,230 historic observations were used to create polygons of the historic eelgrass distribution in NW Kattegat (Figure 1). Cells falling within these polygons were considered suitable habitat and were added to the extant habitat map for use in the biophysical model.

### 2.7 Oceanographic dispersal based on particle modelling

Single‐ and multigeneration dispersal probabilities were estimated with biophysical modelling based on the NEMO‐Nordic (BaltiX) circulation model and the offline Lagrangian particle tracking model TRACMASS (De Vries & Döös, 2001). Virtual particles released in the modelling runs represent eelgrass shoots with spathes containing seeds. NEMO‐Nordic is a regional Baltic/North Sea configuration of the NEMO ocean model (Madec, 2010), with a horizontal resolution of 3.7 km (two nautical miles) and a vertical resolution of 56 layers of variable depth (for details see Hordoir, Dieterich, Basu, Dietze, & Meier, 2013; Moksnes, Jonsson, Nilsson Jacobi, & Vikström, 2014). Tidal harmonics define the sea surface height and velocities at the boundaries, and Levitus climatology defines temperature and salinity (Levitus & Boyer, 1994). The model has a free surface, and the atmospheric forcing is a dynamic downscaling of the ERA40 data set. Runoff is based on climatological data based on a number of different databases for the Baltic Sea and the North Sea.

Velocity fields were updated in the model domain every three hours, and the calculation of particle trajectories was performed with a 15‐minute time step. Particles representing drifting shoots were released from all model grid cells in the Skagerrak–Kattegat that represent the extant or historic distribution of *Z. marina* (Figure 1). Release times spanned July, August and September, with respective proportions of 20, 50 and 30% released particles in each month. Eelgrass reproductive shoots are positively buoyant, and particles in the model drifted in the surface layer (0–2 m). Drift duration was distributed over 5, 10, 20 and 30 days with the proportions 5, 10, 20 and 65% of particles, respectively. *Zostera marina* flowering and detachment periods, as well as duration that shoots stay afloat, were based on empirical field studies along the Swedish Skagerrak coast (Infantes & Moksnes, 2017; Källström et al., 2008). Different drift durations simulate that individual spathes with seeds on the same shoot mature at different times and that some negatively buoyant seeds may be dropped and sink, while the shoot continues drifting (Infantes & Moksnes, 2017). Particle release was repeated for 8 years (1995–2002), representing years with a range of North Atlantic oscillation index values (NAO, Hurrell & Deser, 2010), which is known to correlate well with the variability in circulation pattern. In total, 2.5 million particle trajectories were included. Dispersal probabilities between all sampling sites, over a single generation, were calculated by summing all the trajectories starting in site *i* having end positions within site *j,* normalized by the total number of simulated trajectories from site *i*. We also calculated multigeneration connectivity where stepping‐stone dispersal was allowed over 32 single‐generation dispersal events by multiplication of the single‐generation dispersal matrix with itself 32 times producing connectivity probabilities when summed over all possible dispersal routes (White et al., 2010). Stepping‐stone dispersal was only allowed between grid cells that intersected with the known extant or reconstructed historical habitat distribution (see Figure 1). Stepping‐stone dispersal over 32 generations was considered sufficient to span the approximate spatial scale (~500 km distance) of the model domain. In terms of the temporal scale, 32 generations may represent as little as 32 years when assuming annual sexual reproduction of *Z. marina*, or >1,000 years when assuming high levels of clonal reproduction and clone longevity (Reusch, Boström et al. 1999, Reusch, Stam et al. 1999).

### 2.8 Oceanographic dispersal barrier analysis

We employed a clustering method to identify partial dispersal barriers based on modelled dispersal probabilities in the seascape (Nilsson Jacobi et al., 2012). Only dispersal between areas with present or historic distribution of eelgrass was considered. This theoretical framework finds partially isolated clusters. Identification of clusters is formulated as a minimization problem with a tunable penalty term for merging clusters that makes it possible to generate population subdivisions with varying degree of dispersal restrictions. As the focus was to compare putative dispersal barriers to genetic differentiation of *Z. marina*, the mean connectivity between oceanographic clusters was set low (0.004).

### 2.9 Isolation by “sea distance“ and oceanographic distance

To test for IBD, we correlated genetic distance with ‐sea distance,‐ defined here as the shortest path possible among sampling sites at sea without crossing land. We used the R package *marmap* (Pante & Simon‐Bouhet, 2013) to calculate “sea distance.“ One distance value (between 5‐BO and 6‐NH) had to be adjusted manually to ensure that no land was crossed. Mantel tests were carried out using the R package *ncf* (Bjornstad, 2009) in R 3.2.2. Matrices were resampled 100,000 times and after log_10_ transformation of “sea distance.“

To test for isolation by oceanography (IBO), we correlated genetic distance with minimum oceanographic dispersal probabilities (calculated from the model described above) defined here as oceanographic distance. We used minimum dispersal probability to generate a symmetric matrix of dispersal, because it may arguably be best correlated with (symmetric) geographic and genetic distance (Wrange et al., 2016). We also correlated directional dispersal probabilities with asymmetric (genetic) migration rates in a Mantel test adapted for asymmetric matrices (Matlab 2016, Mathworks Inc). We considered dispersal probabilities for single‐generation‐extant, multigeneration‐extant and multigeneration‐historic distributions. All dispersal probabilities were log_10_‐transformed. As some probabilities were zero, the transformation was performed as follows: log10(single‐generation dispersal matrix + 1e‐10) and log10(multigeneration matrix/historic multigeneration matrix + 1e‐30).

### 2.10 Network analyses

**Figure 2:**
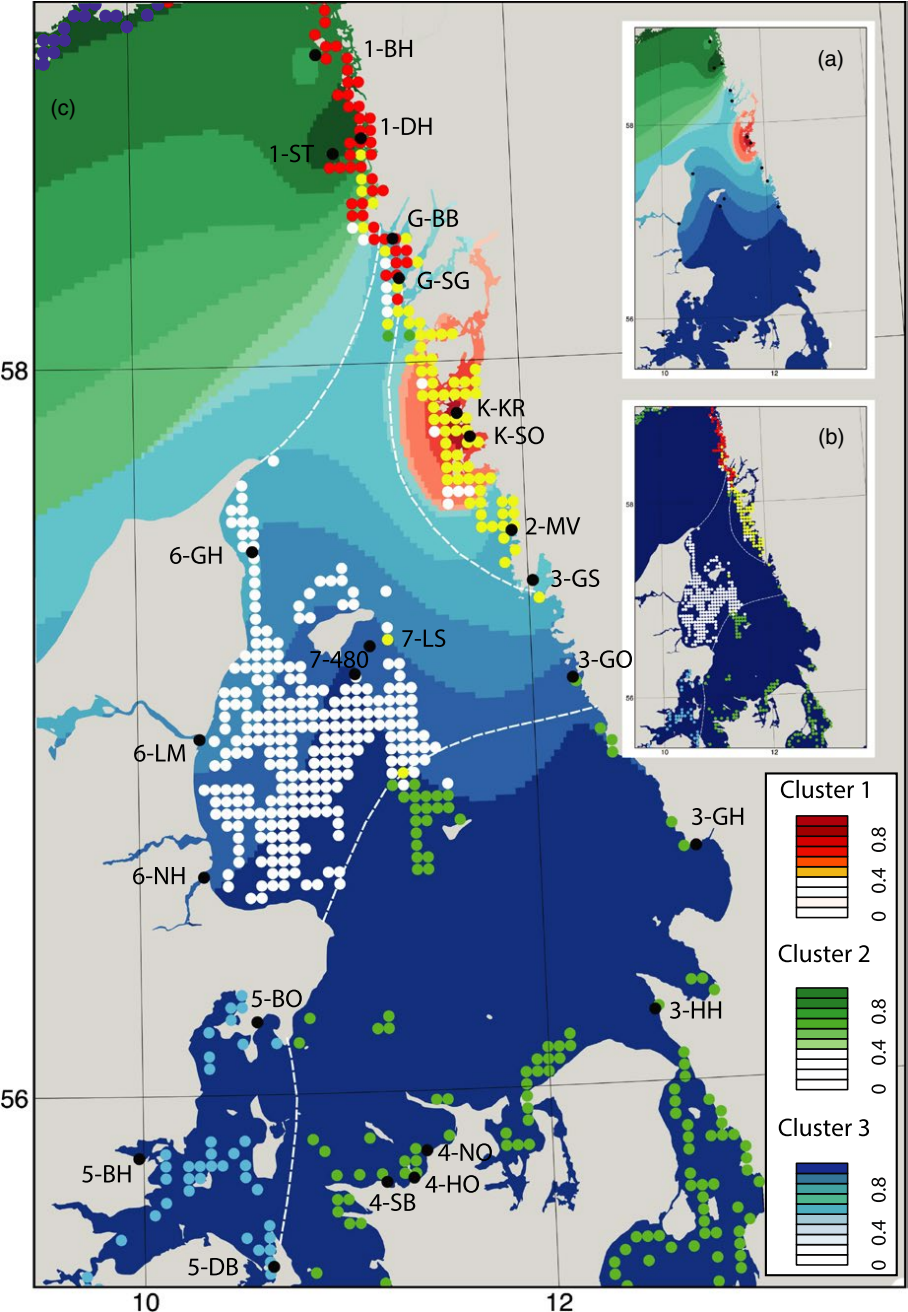
Genetic population structure and oceanographic barrier analysis for the 23 sampling sites of *Zostera marina* in the Skagerrak–Kattegat region of the North Sea. Sampling sites are indicated by black dots with acronyms of the sites as shown in Table 1. (a) Genetic clusters (green, blue and red) show the spatial interpolation of ancestry coefficients (*Q*‐values or proportion of individuals belonging to each cluster) based on the TESS analysis with *K*_max_ = 3; the gradient within each colour indicates percentage of group membership belonging to genetic clusters 1–3 (see inlayed box). (b) The coloured dots (red, yellow, white, green, violet and light blue) represent release points of particles in the oceanographic modelling. The different colours indicate the different oceanographic clusters identified by a clustering method based on modelled multigeneration‐historic dispersal probabilities. Dots with the same colour indicate areas that have an internal connectivity above the dispersal restriction, and the transitions of colours thus indicate partial dispersal barriers. Major barriers among the hydrodynamic clusters are shown with white dotted lines. (c) Superimposed genetic (shown in a) and oceanographic clusters (shown in b) illustrating the good fit between the two analyses, which is further supported by a network analysis in Figure 3

Network analysis is a graphic approach with many applications, one of which is to understand landscape patterns of connectivity and prioritize areas for conservation (Engelhard et al., 2016; and references therein). We used networks to examine connectivity both for genetic (*D*_ps_) and oceanographic distance applied to modelled dispersal probability matrices for single‐generation‐extant, multigeneration‐extant and multigeneration‐historic dispersal probabilities, and to highlight sites that are central to connectivity. Networks were drawn using the R packages *igraph* (Csardi & Nepusz, 2006) and *popgraph* (Dyer, 2014), where nodes represent populations and edges the pairwise distance among populations. Thresholds were chosen systematically following the “intermediate threshold“ method of Greenbaum, Templeton, and Bar‐David (2016). The informative and intermediate thresholds were as follows: *D*_ps_ = 0.18, minimum single‐generation dispersal probability = 2e‐4, minimum multigeneration‐extant dispersal probability = 2e‐14 and minimum multigeneration‐historic dispersal probability = 1e‐12. Use of these thresholds resulted in the loss of five populations from each network, as also seen in the Bayesian clustering analysis (Figure 2). Only edges with genetic distances below the threshold and dispersal probabilities above the threshold are shown.

## 3 RESULTS

### 3.1 Genetic data quality checks and discrimination power

We identified 14 to 40 *MLG*s per population (Table 1), resulting in 756 genets (of 920 ramets) that were used for all further analyses. Locus D2 showed a frequency of null alleles >10% (NaF = 0.115) and was removed from further analyses. For the remaining 21 loci, we tested for HWE and LD. Nine HWE tests per population and locus were significant (1.9%), and significant LD was present in 135 of 4,830 tests across all populations (2.8%) after applying Bonferroni corrections. In both cases, locus GA35 drove most of the significant deviations and was, therefore, removed. Rerunning the analyses on the remaining 20 loci showed only a low percentage of deviations in LD (1.2%) and HWE (1.1%).

The outlier analyses identified several loci to be potentially under balancing and positive selection (Figure S1). As their exclusion did not alter PCAs (Figures S3 and S4), they were retained. A PCA that included additional sites from the Baltic and the North Sea did not reveal any indication that our study area may represent a secondary contact zone between genetically differentiated Baltic and North Sea *Z. marina* meta‐populations (Figure S5).

The probability of identity (PI) by chance for a 20‐locus *MLG* was low ranging from 5.9 × 10^−7^ in population 3‐GH to 3.5 × 10^−10^ in population 2‐MV. The probability for detection of sibs was higher ranging from 1 × 10^−3^ to 6 × 10^−5^. Statistical power simulations in POWSIM of the 20‐locus set indicated a 100% probability of detecting an *F*_ST_ as low as 0.0025, and the α error (false significance) was close to the intended value of 0.05 (Table S3).

### 3.2 Genetic diversity and population differentiation

Genotypic and allelic diversity was found to be high overall (Figure 1 and Table 1) indicating a dynamic and diverse environment characterized by predominant sexual reproduction. One *MLG* was shared among two populations (Gottskär, 3‐GS and Gåsö, G‐SG) separated by 120 km. This is within our estimates for single‐generation northward oceanographic dispersal and reattachment at Gåsö. This genotype has three alleles at three loci not otherwise found at Gåsö. Population differentiation using pairwise shared allele distances (*D*_ps_; 0.09–0.36) or *F*_ST_ (0.01–0.21) was significant (*p* < .05; Table S4 and S5) and consistent with strong overall population structure.

Further characterization of the genetic population structure started with a PCA that generated a horseshoe‐shaped cloud of apparently little differentiation based on the first two axes (Figure S2) and even less on the third axis (not shown). Such a pattern is expected under scenarios in which allele frequencies are locally correlated and thus covariance is decaying with geographic distance in an IBD pattern (Frichot, Schoville, Bouchard, & François, 2012).

Continuing on, the spatial Bayesian analysis in TESS suggested genetic population subdivision into three clusters (*K*_max_ = 3, Figure 2), which was very similar to the *K* = 3 scenario of Structure (Figure S6 and S7). The three northern populations on the west coast of Sweden form a small (green) cluster that exhibits low allelic richness. The small (red) genetic cluster consisting of the two sites South Kråkerön (K‐KR) and North St. Överön (K‐SO) is located in the Marstrand area. The large (blue) cluster extends over the entire Kattegat and includes all Danish sites. Additionally, a gradient from south to north is evident in the blue contours of Figure 2a, where the more northern sampling sites represent admixtures with the red clusters (see also Figure S7). Under the two‐cluster partition suggested by Structure, the two smaller TESS clusters 1 and 2 are depicted as one.

### 3.3 Directional migration rates

Directional migration based on the genetic data was estimated in two ways. Based on DivMigrate (Table S8 and Figure S8a), directionality was stronger from south to north with eight sites identified as sources and five identified as sinks. The genetic assignment test based on GENECLASS2 (Table S9 and Figure S8b) identified 31 first‐generation migrants, of which only seven could be assigned to other sampling sites (Table S9). Here, directionality was predominantly south to north and west to east. Both methods confirm that long‐distance dispersal occurs.

### 3.4 Oceanographic dispersal based on particle modelling

The biophysical particle modelling indicated dispersal up to 200 km in a single generation, consistent with other estimates for *Z. marina* (Harwell & Orth, 2002; Källström et al., 2008), and more than 300 km when allowing stepping‐stone dispersal over multiple generations. Few particles (modelled seeds) dispersed between sample sites during a single generation, although local retention within the same sampling occurred (Figure S9). The most northern sites (names starting with 1 and G) received particles from many other meadows; sites from the sampling areas 6 and 7 supplied particles to most other sites.

Probabilities for multigeneration oceanographic dispersal based on the present‐day distribution were much lower than those based on single generations, but there were nonzero probabilities of dispersal among all sampling sites (Figure S10). The site 3‐HH was the best source for particles, while 1‐ST and 6‐LM supplied few particles to other sites. Inclusion of the historic distribution of *Z. marina* (Figure 1) in multigeneration dispersal modelling only slightly changed the overall picture, but with important differences at certain sites. For instance, 6‐LM and G‐SG acted as much stronger sources and 6‐GH, 6‐LM, 6‐NH and 4‐HO received considerably more particles in the past (Figures S10–S12). Notably, the lowest dispersal probabilities increased by two orders of magnitude compared to multigeneration dispersal based on the present‐day distribution alone.

### 3.5 Oceanographic dispersal barrier analysis

At the chosen threshold, the minimization algorithm applied to the multigeneration dispersal matrix (including the historic habitat) generated six oceanographic clusters with partial barriers among them (Figure 2b). Connectivity within oceanographic clusters was approximately 100 times greater than among clusters. Four barriers were identified (Figure 2): (i) at 58°N, which spatially coincides with the green genetic cluster and the division between the Kattegat and Skagerrak; (ii) a barrier along the Swedish Kattegat coast encompassing the red genetic cluster; (iii) at 57°N roughly following the gradient from “pure“ to high genetic admixture observed within the blue cluster; (iv) a barrier across the south‐west corner of the Kattegat. This last barrier was not reflected in the genetic cluster analysis, but the asymmetric migration analysis also indicated low gene flow in this area (Figure S8).

### 3.6 Isolation by “sea distance“ and oceanographic distance

Geographic distance defined as “sea distance“ (see Material and Methods) ranged from *~*10 to ~400 km. A significant pattern of isolation by “sea distance“ (IBD) with genetic differentiation (*D*_ps_) is evident (Table 2). While oceanographic connectivity based on single‐generation dispersal probability was not correlated with the genetic differentiation measure *D*_ps_, multigeneration‐extant and historic dispersal were strongly correlated (three different scenarios of IBO). IBO was strongest for the dispersal probability based on the historic distribution of *Z. marina,* which reached a correlation coefficient as high as −0.59 (Table 2). Patterns of *F*_ST_‐related genetic indices were similar (not shown). IBO was also observed for the correlation between genetic asymmetric migration rates and the directional dispersal probabilities (Table 2). For both genetic differentiation indices, correlation coefficients are much higher for multigeneration compared to single‐generation dispersal probabilities. This indicates that stepping‐stone dispersal over several generations can explain genetic differentiation better than single‐generation dispersal probability, which is limited by geographic distance (Table 2). Correlations are further improved when considering the historic distribution of *Z. marina* (Table 2).

**Table 2:** Results of Mantel tests between log_10_‐transformed “sea distance“ or dispersal probabilities (see Material and Methods) and a genetic differentiation matrix based on the proportion of shared alleles (*D*_ps_) or asymmetric migration rates based on *G*_ST_ and calculated with DivMigrate (asymm. mig.)

	“sea distance“		Single‐generation dispersal probability		Multigeneration dispersal probability		Historic multigeneration dispersal probability	
	*D*_ps_	Asymm. mig.	*D*_ps_	Asymm. mig.	*D*_ps_	Asymm. mig.	*D*_ps_	Asymm. mig.
Corr	.31	na	−.1	.19	−.31	.34	−.59	.39
*p*	**1E‐04**	na	0.106	**0.004**	**2.9E‐05**	**4.2E‐05**	**1E‐05**	**3E‐04**

All but the correlation between single‐generation dispersal probability and *D*_ps_ is significant (bold). Note that a negative correlation is expected between *D*_ps_ and minimum dispersal probability, because sites with a high probability of dispersal between them are expected to show low genetic differentiation, whereas asymmetric migration rates are expected to be positively correlated with dispersal probability.

### 3.7 Network analyses

**Figure 3:**
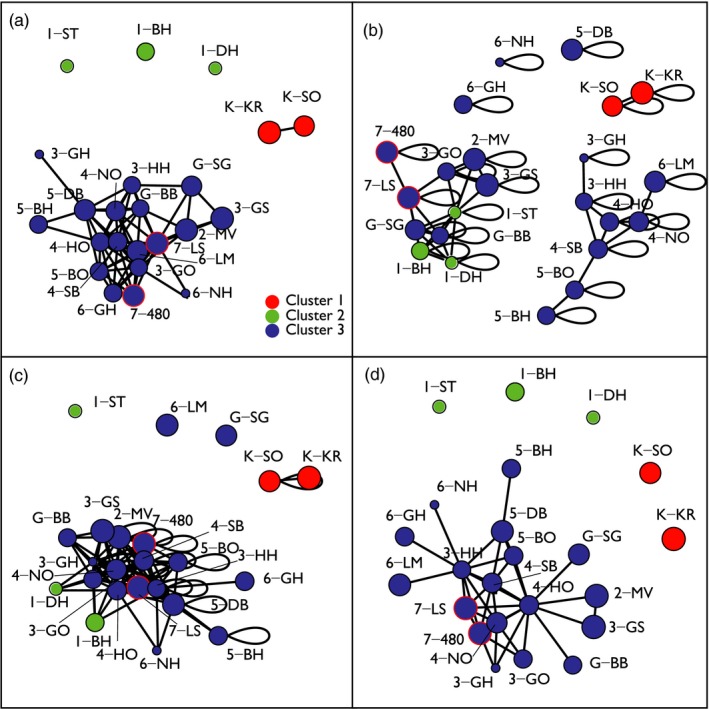
Genetic distance and oceanographic distance networks constructed for the 23 sampling sites of *Zostera marina* in the Skagerrak–Kattegat region of the North Sea. (a) genetic distance (shared alleles, *D*_ps_), (b) oceanographic distance, minimum single‐generation dispersal probability, (c) oceanographic distance, minimum multigeneration‐extant dispersal probability and (d) oceanographic distance, minimum multigeneration‐historic dispersal probability. The colour of nodes matches the clusters identified by the TESS analysis (Figure 2), and the size of a node represents the standardized allelic richness found at the site. The two Læsø Island sites (7‐LS and 7‐480) are encircled in red to highlight their central position

The four network analyses in Figure 3 were in overall good agreement with the TESS and Barrier analyses in Figure 2. In the network based on the genetic differentiation matrix *D*_ps_, all populations from the big blue cluster formed one large network, while the populations from the green and red clusters fell out of this network (Figure 3a). The network drawn for minimum single‐generation dispersal probability (Figure 3b) was the least similar to the TESS picture (Figure 2a). In general, this network showed a more stepwise connectivity pattern among populations from the same or adjacent sampling areas, reflecting that dispersal probability is limited by geographic proximity at the assessed spatial scale. In contrast, the network based on minimum multigeneration (32 generations and taking into account the extant *Z. marina* distribution) was similar to the TESS analysis and the network based on genetic distance (Figure 3c). The network based on multigeneration‐historic dispersal probability was almost identical to the TESS analysis and the genetic network based on *D*_ps_ (Figure 3d).

The network analysis also allowed visualization of populations central to connectivity (Greenbaum & Fefferman, 2017; Rozenfeld et al., 2008). The oceanographic and genetic network analyses indicated that meadows from south‐western and central Kattegat (sampling areas 4, 5 and 7) are central to connectivity (Figure 3). The offshore populations from the island Læsø (7‐LS and 7‐480) are of greatest interest as they are located in an area of historically extensive eelgrass meadows (and still retain high allelic richness; Figure 1).

## 4 DISCUSSION

### 4.1 Distribution of genetic diversity of Z. marina in the Skagerrak ‐Kattegat

Our initial hypothesis of reduced genotypic and allelic diversity as a consequence of the massive losses and fragmentation over the past century was not borne out. Rather, the Skagerrak–Kattegat region harbours some of the highest diversity for *Z. marina* in Europe (J.L. Olsen, unpublished data). Interestingly, the highest values of allelic richness are found in the centre of the Skagerrak–Kattegat region around the Læsø Islands (7‐LS, 7‐480), where eelgrass was historically abundant (Figure 1b). These observations are consistent with probable glacial refugia (Maggs et al., 2008) and the original postglacial colonization of the nascent North Sea basin (Hewitt, 2000; Maggs et al., 2008; Olsen et al., 2004) including the Skagerrak–Kattegat, when the current system was established *ca*. 8,000 years ago (Gyllencreutz, Backman, Jakobsson, Kissel, & Arnold, 2006). At that time, the Baltic was still an isolated, freshwater ice lake and colonization of both areas came most likely from the south (Ireland, Brittany, Iberian tip), although a high North refugium in northern Norway cannot be ruled out for macrophytes in general (Coyer et al., 2011; Maggs et al., 2008; Olsen et al., 2013). No evidence for a secondary contact zone, commonly observed to coincide with biogeographic regions (Gagnaire et al., 2015), was evident between genetically differentiated Baltic and North Sea populations and the Skagerrak–Kattegat (see Figure S5).

### 4.2 Population genetic structure and connectivity

The 23 sampling sites form three distinct genetic clusters based on the TESS analysis (Figure 2). The small (red) genetic cluster consisting of the two sites, South Kråkerön (K‐KR) and North St. Överön (K‐SO), is located in the Marstrand area, which has lost an estimated 93% of meadows since the 1980s and losses continue (Moksnes et al., 2016). Although these two sites are genetically isolated from the other clusters, they exhibit high allelic diversity. Gene flow to these sites may be provided from small fragmented eelgrass beds that are still found in the Marstrand area (currently under investigation). Each of the three clusters is further characterized by strong population genetic structure among all sampling sites and few first‐generation migrants (Table S5). This is typical for seagrasses and caused by one or more of the following factors: partial clonality (Olsen et al., 2004), a larger role for mutations over migration due to the longevity of clones (Arnaud‐Haond et al., 2014), sporadic recruitment (Becheler, Diekmann, Hily, Moalic, & Arnaud‐Haond, 2010), founder‐takes‐all recruitment (Waters, Fraser, & Hewitt, 2013) or stochasticity of dispersal (Kendrick et al., 2012).

Dispersal among populations was further explored with the biophysical particle modelling. Single‐generation dispersal explains the differentiation of the small genetic cluster in the Marstrand area (red cluster in Figure 3b) and is significantly correlated with asymmetric migration rates—but not with genetic differentiation (*D*_ps_; Table 2). Multigeneration dispersal explains a higher proportion of genetic differentiation and asymmetric migration rates (Table 2), and long‐distance connectivity increases (Figure 3c). Inclusion of the historic distribution in the multigeneration model results in an almost perfect recovery of the genetic clusters (Figure 3a,d), and both *D*_ps_ and asymmetric migration rates have an improved fit with this measure (Table 2). Overall, this assessment with presumably neutral genetic markers suggests that the processes of migration and genetic drift explain a large part of the observed genetic population structure, but adaptation to local physical parameters could be an additional explanation.

One major genetic and oceanographic break observed is located approximately at the border of the Kattegat and Skagerrak and is confirmed by the few previous studies of population genetic structure in the Skagerrak and Kattegat, e.g., for herring (Lamichhaney et al., 2012), harbour porpoise (Lah et al., 2016) and cod (Barth et al., 2017). A particularly relevant study of the macroalga *Saccharina latissimi*, which also shows exclusively passive dispersal, indicates a similar genetic break between the Kattegat and Skagerrak (Moller Nielsen et al., 2016). The other important genetic and oceanographic break we observe is located in the Marstrand area. This has not been previously reported—probably due to lack of geographically detailed sampling for genetic studies in the area. The only studies in the Skagerrak and Kattegat that used both genetic and biophysical methods found high correlations between gene flow and oceanographic connectivity for diatoms (Godhe et al., 2013), while the correlation was lower for actively moving cod (Barth et al., 2017).

The network analysis also allows visualization and identification of populations central to connectivity (Greenbaum & Fefferman, 2017; Rozenfeld et al., 2008). The oceanographic and genetic network analyses indicate that meadows from south‐western and central Kattegat (sampling areas 4, 5 and 7) are central for connectivity (Figure 3). The offshore populations from the island Læsø (7‐LS and 7‐480) are of greatest interest as they are located in an area of historically extensive eelgrass meadows (and still retain the phylogeographic footprint of high allelic richness; Figure 1). Their central position and node size within the networks indicate their importance as stepping stones between the Skagerrak and Kattegat as well as between Denmark and Sweden.

### 4.3 Comparison of connectivity measures and temporal scales

The best fit among Mantel tests was obtained between genetic distance based on the proportion of shared alleles (*D*_ps_) and multigeneration‐historic dispersal, explaining ~40% of genetic variability (Table 2). This metric takes into account oceanographic dispersal distance, stepping‐stone dispersal over multiple generations and historic habitat continuity. IBO that incorporates oceanographic distance and habitat discontinuity, and/or stepping‐stone dispersal, was able to achieve similarly high correlations for giant kelps and fucoid macroalgae that also disperse by rafting (Alberto et al., 2011; Buonomo et al., 2017). Thus, support is mounting that IBO is a better approach than IBD to explain genetic structure and gene flow. However, the correlation between genetic differentiation and single‐generation oceanographic dispersal performs worse than classical IBD. Asymmetric migration, calculated with DivMigrate, correlates more strongly than *D*_ps_ with both single‐ and multigeneration‐extant dispersal probability, but not with multigeneration‐historic dispersal. This indicates that this measure of asymmetric migration is capable of capturing more recent migration rates (Sundqvist et al., 2016). This measure is relatively new, and to our knowledge, we test and show here for the first time that this metric has indeed a better fit with dispersal probability on shallow time‐scales, as would be expected (Sundqvist et al., 2016). The good fit of both genetic measures and oceanographic connectivity further indicates that genetic structure of eelgrass in the Skagerrak–Kattegat is mainly driven by migration and genetic drift—and not selection.

Despite the documented high loss of eelgrass meadows in the area, the effect is not visible in the levels of genetic diversity and differentiation. Thanks to the availability of historic distribution data, we were able to compare modelled connectivity of the extant eelgrass beds with the historic distribution of ~100 years ago. As would be expected, the loss of the historic meadows has resulted in some changes in the probability of dispersal and the network structure. For example, Limfjord (6‐LM) and Gåso (G‐SG) have become oceanographically isolated over the last century (Figure 3c,d), but this is not visible (yet) in the genetic structure (Figure 3a). This genetic memory or “ghost of dispersal past“ (Benzie, 1999) reflects distribution and connectivity of *Z. marina* of at least 100 years ago instead of the current (decades) distribution. In fact, it may even reflect the “ghost of original colonization“ after the last glacial maximum. Such mismatches have often been observed and explained by a time lag between current demographic processes and population genetic structure (Epps & Keyghobadi, 2015; and Jahnke, Olsen, & Procaccini, 2015 for seagrasses) and/or high temporal genetic stability of genetic diversity measures (Reynolds et al., 2017).

### 4.4 Complementary value of genetic and biophysical models

Cross‐validations of the genetic and oceanographic modelling data show good agreement and provide different insights into the structure and connectivity of populations. The genetic survey integrates over many gene‐flow mechanisms and captures regional population history through deep time. In addition, diversity metrics and population differentiation, as well as inferences about demography, can only be determined with genetic data. In general, genetic methods are less well suited for inferring the spatial component and directionality of dispersal.

In contrast, biophysical models provide insights about the generational time depth of dispersal and the shaping of populations with respect to barriers and circulation patterns. Although single‐generation dispersal may be a weak predictor, the ability to simulate a range of generational time depths through stepping‐stone simulations is a distinct advantage. When historical distribution records are also available, as is the case here, predictions of where populations should or could persist become very powerful. Biophysical models also offer better spatial coverage than is feasible with most genetic sampling efforts. The main shortcoming of biophysical models is that they say nothing about demographic history, adaptive potential or genetic health of the species in question. In terms of resource investment, initial front‐end development of suitable oceanographic models is both time‐consuming and cost‐intensive, and limited to the specific region of interest. In the absence of such oceanographic models, genetic surveys remain the best alternative. Microsatellite markers are available for many seagrass species, and an assessment such as the one reported here is standardized and easy to perform.

The added value of the dual approach further strengthens conservation planning and eventual monitoring of a particular management plan, because it is possible to rerun a biophysical model with different data and under different scenarios to reflect adaptive management (e.g., McCook et al., 2010). The results of the two approaches to connectivity may also be used to rank sites, for instance according to their connectedness, whether they act as sources or sinks or their level of diversity (Jonsson, Nilsson Jacobi et al., 2016). Such results could then be used by conservation managers in spatial planning programmes such as Marxan and Zonation for prioritization in large‐scale conservation efforts (Delavenne et al., 2012).

### 4.5 Implications for management

Understanding spatial population structure and identifying areas with restricted as well as excellent connectivity are essential in conservation management. Here, both the genetic and hydrodynamic connectivity assessments identified dispersal barriers, creating distinct clusters that could serve as *management units* (Palsbøll, Bérubé, & Allendorf, 2007), which should be managed separately to ensure long‐term persistence and protection of genetic diversity (Allendorf et al., 2013). While a genetic and oceanographic barrier is evident between the Kattegat and Skagerrak, no such break is visible between Denmark and Sweden. Thus, it is important to assess whether existing or proposed MPAs in the study area constitute functional networks within each of the three genetically distinct clusters and their biophysical barriers also across countries. For instance, all *Z. marina* meadows within the large cluster covering most of the Kattegat could be managed as a single unit within, for example, an MPA network, as replenishment from one site to another can be expected. However, the two sites that have become oceanographically isolated (6‐LM and G‐SG) will require further local management. Likewise, it is critical to protect the small meadows off the Island Læsø (7‐LS and 7‐480), remnants of the large historic offshore population that appear key for connectivity (Figure 3). Fortunately, the meadows off Læsø are presently included in a *ca*. 1,000 km^2^ large Natura 2000 site that includes protection of shallow water soft‐sediment habitats (Moksnes et al., 2014). However, dramatic improvements of the environmental conditions in, for example, water clarity would be required for recovery towards the historic distribution in offshore Kattegat where the maximum depth distribution of eelgrass has decreased by >50% (Boström et al., 2003). It is also important to ensure that MPAs provide the intended protection to habitats and biodiversity (Almany et al., 2009). Along the Swedish coasts, for example, small‐scale destruction of eelgrass meadows for construction of piers and marinas is high and occurs even inside protected areas (69% of the studied cases within eelgrass meadows were approved for construction; Eriander, Laas, Bergström, Gipperth, & Moksnes, 2017). Hence, there is an urgent need to review regulations and management of existing MPAs.

From a local management perspective, one of the key findings is the low connectivity into and out of the Marstrand area (red cluster 1) in the Swedish Kattegat, where major losses of eelgrass have occurred and continue to occur. The oceanographic isolation could be related to two large rivers that enter the Kattegat just south of this area and may create a dispersal barrier. The genetic isolation indicates that natural replenishment from outside is unlikely, making protection of the remaining *Z. marina* beds crucial. The high allelic diversity suggests that the losses have not yet negatively affected fitness and that local meadows constitute good donor material for restoration (e.g., Reynolds et al., 2012), while transplantation from other areas should be avoided (e.g., Kettenring, Mercer, Reinhardt Adams, & Hines, 2014). In fact, this study shows that genetic diversity and connectivity of *Z. marina* in the Skagerrak–Kattegat seem to be generally in a healthy state, but assessment such as this, in addition to assessments on a more local scale, can highlight vulnerable sites or could be used as baselines for tracking future changes.

The historic loss in the Skagerrak–Kattegat is possibly the largest reported seagrass loss in the world (P.‐O. Moksnes, unpubl.). Despite protection by international conventions and directives, and large MPA networks (covering approximately 15% of the Skagerrak–Kattegat; Moksnes et al., 2014), recovery has been very limited, and losses continue (Boström et al., 2014; Moksnes et al., 2016). Thus, protection from physical impacts within MPAs may not be sufficient to ensure persistence and recovery of *Z. marina,* but more and new measures are needed to improve environmental conditions. In addition to increased efforts to reduce nutrient input to coastal waters, management should consider measures to enhance depleted populations of large predatory fish that would restore the trophic structure of coastal ecosystems (Östman et al., 2016), measures that can break self‐generating feedback mechanisms such as sediment resuspension that lock the system in a turbid state (Maxwell et al., 2016; Nyström et al., 2012), and eelgrass restoration to facilitate a natural recovery of lost meadows (van Katwijk et al., 2016), including compensatory mitigation of eelgrass lost or damaged during, for example, coastal exploitation (Moksnes et al., 2016).

## 5 CONCLUSIONS

Our analysis supports the notion that passive rafting of flowering shoots by oceanographic currents influence patterns of gene flow of *Z. marina* in the Skagerrak–Kattegat and is the main driver of observed population genetic structure and meta‐population dynamics. We show that the meta‐population is driven by stepping‐stone dispersal over many generations and that current genetic differentiation is best explained by connectivity considering the historic *Z. marina* distribution. This “;ghost of dispersal past“ is also evident in the distribution of allelic richness, where highest diversity is found in the Læsø Island area, where major historic losses occurred. Using two complementary methods to assess connectivity enabled us to investigate and compare dispersal and migration patterns at different temporal scales. In this study, we found strong concordance among the two methods in detecting sources, sinks and connectivity patterns. This information can be used to pinpoint areas where local protection is necessary or where populations could/should be managed in a network approach for MPAs. The temporally more dynamic oceanographic modelling was also able to highlight areas where connectivity has become limited over the last decades. Such information is additionally helpful for marine spatial management to pinpoint geographic areas where there is a need to improve environmental conditions. The large geographic scale study presented here forms a framework for future detailed assessments of connectivity and genetic diversity on smaller scales within the coastal archipelagos and fjords. Such multiscale information should aid managers at the local, national and international levels in marine spatial planning, for example, for the identification of hubs, and important extant or historic source meadows that should be targeted for protection, or key areas for restoration.

## DATA ARCHIVING STATEMENT

The matrix of microsatellite genotypes and the oceanographic matrices of dispersal probability can be found on Dryad: https://doi.org/10.5061/dryad.2139f.
