# Supplementary material for: Seascape genetics and biophysical connectivity modelling support conservation of the seagrass Zostera marina in the Skagerrak–Kattegat region of the eastern North Sea
Source: Evol Appl. 2018 Jan 26;11(5):645–61. doi: 10.1111/eva.12589 (PMC5979629; doi:10.1111/eva.12589)
Supplement: Supplementary file 1 [file EVA-11-645-s001.docx]

**Supporting Information**

**Seascape genetics and biophysical connectivity modelling support conservation of the seagrass *Zostera marina* in the Skagerrak-Kattegat of the eastern North Sea**

**M. Jahnke, P.R. Jonsson, P.-O. Moksnes, L.-O. Loo, M. Nilsson Jacobi and J.L. Olsen**

**Table of contents**

| **Genotyping** |  |
| --- | --- |
| Table S1 Primer details | 2 |
| Table S2 PCR multiplex reactions | 3 |
| **Data diagnostics and quality checks** |  |
| Fig. S1 Outlier analysis | 4 |
| Fig. S2 PCA | 5 |
| Fig. S3 Evaluation of outliers | 6 |
| Fig. S4 Evaluation of outliers | 7 |
| Fig. S5 PCA to test for secondary contact zone | 8 |
| Table S3 Power simulation of loci | 8 |
| **Genetic distance matrices** |  |
| Table S4 Shared allele distance *D*_ps_ | 9 |
| Table S5 Weir & Cockerham *F*_ST_ | 11 |
| Table S6 Hedrick’s *G’*_ST_ | 13 |
| Table S7 Jost’s *D*_EST_ | 14 |
| **Population structure analyses** |  |
| Fig. S6 DIC and deltaK analyses | 15 |
| Fig. S7 Comparison of TESS and Structure | 16 |
| **Directional migration** |  |
| Table S8 Asymmetric migration DivMigrate | 18 |
| Table S9 Assignment test | 19 |
| Fig. S8 Visualisation of asymmetric dispersal | 21 |
| **Oceanographic dispersal probability** |  |
| Fig. S9 Heat map of single generation dispersal probability | 22 |
| Fig. S10 Heat map of multi-generation connectivity | 23 |
| Fig. S11 Heat map of historical multi-generation connectivity | 24 |
| Fig. S12 Heat map of change in multi-generation connectivity | 25 |
| **References** | 26 |

**Table S1** Primer details for the 22 microsatellite loci used in the analysis of the 23 *Zostera marina* meadows in the Skagerrak - Kattegat. Names appearing in parentheses are the original names of EST-library-derived loci from Keil (2011). The last column (Multi-plex) indicates the primer group (4, 5, B, C or D) in which primers were multiplexed.

| Name | Forward | Reverse | Label for F | Multi-plex |
| --- | --- | --- | --- | --- |
| GA2* | TGAAGAAATCCCAGAAATCCC | AGACCCGTAAAGATACCACCG | FAM | 4 |
| GA23** | GGCAGCGATCTAATAACAATTAAGG | ACGTCACATCTTTTCACGACC | HEX | 4 |
| GA35*** | TCTTGGGCTTTTAATTAGCG | AAAGAGAGACCTAAAGATATGGGC | HEX | 4 |
| H1 (CL202Contig1) | TTGAAAAGATTAATTATTGGTGGTG | TCAAGTCCGGATAAATTCGAT | FAM | 4 |
| A3 (ZMC19017) | TCGTCGAGAAAGAGGAGGAA | TGTTCTGATTCCGTTCTCCA | FAM | 5 |
| GA12 | CGTTCATCTTGTCCTCGTCC | TTTCATTTCCATTTCCCACC | FAM | 5 |
| GA16 | AGAAACCCTAATGTGATGAAATG | TGTTGGTTAATTCTCTTCTAATCTTG | NED | 5 |
| GA17D | TTTTCATTTATCCAATAGTTTGCC | GATTCTCATCGGAGATTGAGG | FAM | 5 |
| GA19 | CCCAAGAAATATAAAATCGGGG | CTTCTCCTTCCGCCGCTAC | HEX | 5 |
| GA20 | TGGAAGGAGTTTCGATGTATCC | GGGAGATTTGCAGTGTAGAATTTAG | FAM | 5 |
| A2 (CL32Contig2) | AATCTGTTGCCACGAAGGAG | TCACCTTCATCAAGCAGTCG | FAM | B |
| B1 (CL853Contig1) | CATTCCATTCAAGAGCAGCA | CAACAAATCAATCAATCATTCACTC | FAM | B |
| B2 (CL679Contig1) | ATAAAAACCGGCCTGATCG | CACACACACAGACGATCGAA | HEX | B |
| B3 (ZMC06073) | CGAATCCTCCTGCGTCTTT | ACGCACCGGATTTTATGCT | NED | B |
| C1 (ZMC02023) | TCGTTGGATACTGCACTTCTC | GAATCCAACCAATTATTTAAATACC | NED | C |
| C2 (ZMC19089) | AGTGAAAAAACAAAGAAAGAAAGAGAAC | CGTCGTCAGGTAGGCTCAA | HEX | C |
| C3 (ZMC12075) | CCTCTTTTTTCCTCTCTCTCTCTCT | CTTCTGCGAATGATGCCATA | HEX | C |
| C4 (CL734Contig1) | TGGTCTTCTTCACTCGCTCA | AGCGACGATTCTTCAGCATT | NED | C |
| D1 (ZMC13053) | CCCCATCTTTTGAGTTTGGA | TCATCATTTCTTGCAATTTGAATC | FAM | D |
| D2 (CL412Contig1) | GTACACCCGTTGCGTTCATT | GATTCCGTAGACTTGCGTCTG | HEX | D |
| G4 (ZMF02381) | GTGCAGGCGATCGAGTTATC | AAATTCGAGCTCTCAACTTCAA | HEX | D |
| K1 (ZMC05062) | GAAGCCAACTTAATTCAACATCG | TTAATATAAATCCGAGACACAGACTC | HEX | D |

Note: Alternative primer names have appeared in some of our publications. Please check the actual sequences to make sure if you are combining data.

*GA2 as CT3 ([Olsen *et al.* 2014](#_ENREF_12)), as CT2 ([Olsen *et al.* 2013](#_ENREF_13))

**GA23 as CT23

***GA35as CT35

**Table S2** PCR multiplex reactions used in the analysis of 23 *Zostera marina* meadows. Volumes for primers are based on 20µM working solutions for primers starting with GA, and 5µM working solutions for all remaining primers. MM, master mix of the Qiagen Kit Type-IT®. For further details see Materials and Methods.

| **4-plex** |  |  | **5-plex** |  |  | **B** |  |  | **C** |  |  | **D** |  |
| --- | --- | --- | --- | --- | --- | --- | --- | --- | --- | --- | --- | --- | --- |
|  | add µL |  |  | add µL |  |  | add µL |  |  | add µL |  |  | add µL |
| 2xMM | 3.175 |  | 2xMM | 3.175 |  | 2xMM | 3.175 |  | 2xMM | 3.175 |  | 2xMM | 3.175 |
| PrimGA2-F | 0.15 |  | GA12-F | 0.05 |  | B1-F | 0.025 |  | C1-F | 0.05 |  | D1-F | 0.025 |
| PrimGA2-R | 0.15 |  | GA12-R | 0.05 |  | B1-R | 0.025 |  | C1-R | 0.05 |  | D1-R | 0.025 |
| PrimGA23-F | 0.05 |  | GA19-F | 0.05 |  | B2-F | 0.025 |  | C2-F | 0.05 |  | D2-F | 0.025 |
| PrimGA23-R | 0.05 |  | GA19-R | 0.05 |  | B2-R | 0.025 |  | C2-R | 0.05 |  | D2-R | 0.025 |
| PrimGA35F | 0.25 |  | GA20-F | 0.1 |  | B3-F | 0.025 |  | C3-F | 0.025 |  | G4-F | 0.01 |
| PrimGA35R | 0.25 |  | GA20-R | 0.1 |  | B3-R | 0.025 |  | C3-R | 0.025 |  | G4-R | 0.01 |
| H1 | 0.025 |  | GA17-D-F | 0.15 |  | A2-F | 0.025 |  | C4-F | 0.025 |  | K1-F | 0.01 |
| H1 | 0.025 |  | GA17-D-R | 0.15 |  | A2-R | 0.025 |  | C4-R | 0.025 |  | K1-R | 0.01 |
| water | 1.125 |  | GA16-F | 0.15 |  | water | 1.875 |  | water | 1.775 |  | water | 1.935 |
| DNA | 1 |  | GA16-R | 0.15 |  | DNA | 1 |  |  |  |  | DNA | 1 |
|  |  |  | A3-F | 0.025 |  |  |  |  |  |  |  |  |  |
|  |  |  | A3-R | 0.025 |  |  |  |  |  |  |  |  |  |
|  |  |  | water | 1.025 |  |  |  |  |  |  |  |  |  |
|  |  |  | DNA | 1 |  |  |  |  |  |  |  |  |  |
| **TOTAL**  **Reaction Vol.** | **6.2** |  |  | **6.2** |  |  | **6.2** |  |  | **6.2** |  |  | **6.2** |

**Fig. S1** Outlier analysis of the 23 *Zostera marina* meadows in the Skagerrak - Kattegat with (a) Lositan and (b) BayesScan. For Lositan the analysis based on and infinite alleles model (IAM) is shown. The two methods differ in their approach as follows: Lositan identifies outliers with higher than neutral heterozygosity conditioned on the fixation index *F*_ST_ ([Antao *et al.* 2008](#_ENREF_1)); BayesScan uses posterior distributions generated by MCMC to identify whether a model including selection is more likely than a model without selection ([Foll & Gaggiotti 2008](#_ENREF_6)). In Lositan, simulations were run for 50,000 iterations, with a 95% confidence interval, using the options “neutral mean *F*_ST_”, “force mean *F*_ST_”, a subsample size of 40 individuals and the infinite allele model. In BayeScan, default settings were used, which result in the same probability threshold as used for Lositan. We used the R script provided by Foll & Gaggiotti (2008) to analyse if any loci deviated significantly from expectation under neutrality and for plotting the posterior distribution.


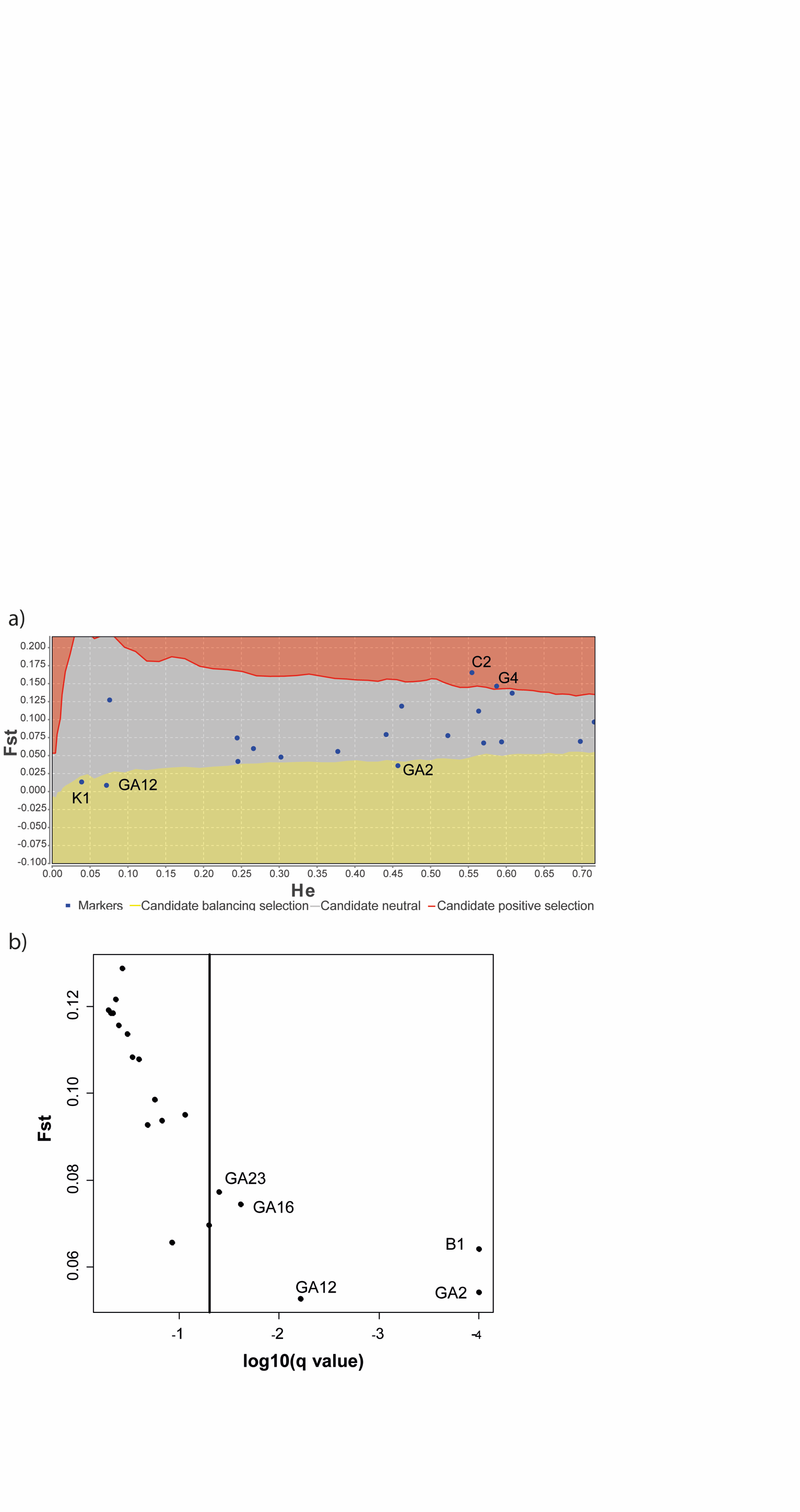


**Fig. S2** Principal component analysis of the 23 *Zostera marina* meadows in the Skagerrak – Kattegat region of the North Sea. The first two axes capture a horse-shoe shaped pattern of differentiation with high overlap among most sites. Each dot represents an individual contained into populations by an ellipse of the population specific colour. Each ellipse represents 95 % of the inertia of the corresponding group. Shown in the insert box are the eigenvalues for the principal components (PCs), with the first two PCs shown in the PCA highlighted in black, which together explain 41% of the variance.

**
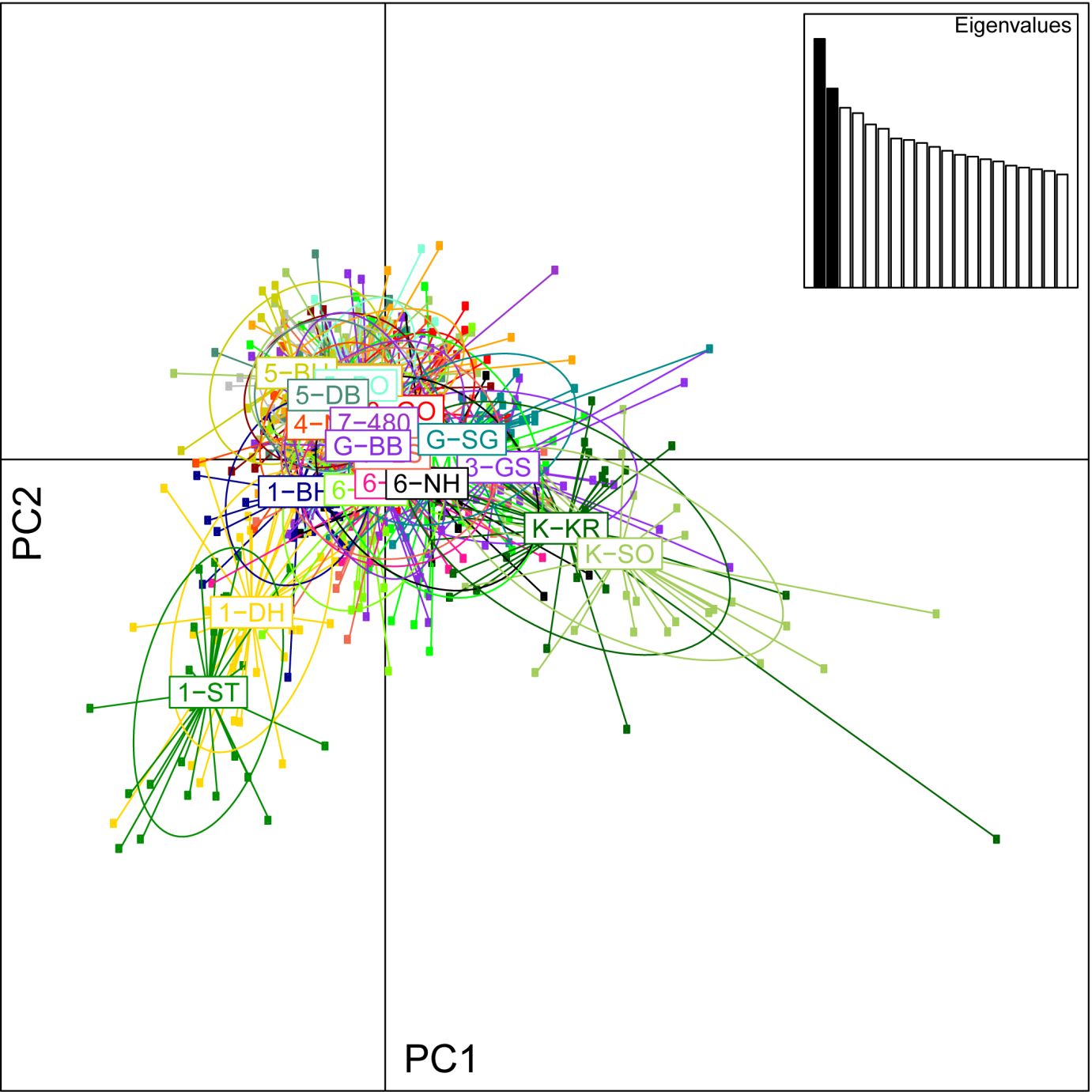
**

**Fig. S3** Evaluation of outliers. PCA analysis without the 2 loci detected to be **under balancing selection** (GA2 and GA12, Fig. S1) of the 23 *Zostera marina* meadows in the Skagerrak - Kattegat. PCA is a mathematical representation of orthogonally partitioned variances without any underlying assumptions from population genetics theory and therefore useful for a general view of the data structure as well as possible technical artefacts. The two loci putatively under balancing selection do not change the shape of the PCA based on all loci (shown in Fig. S2). Each dot represents an individual contained into populations by an ellipse of the population specific colour. Each ellipse represents 95 % of the inertia of the corresponding group. Shown in the insert box are the eigenvalues for the principal components (PCs), with the first two PCs shown in the PCA highlighted in black, which explain 46% of the variance.


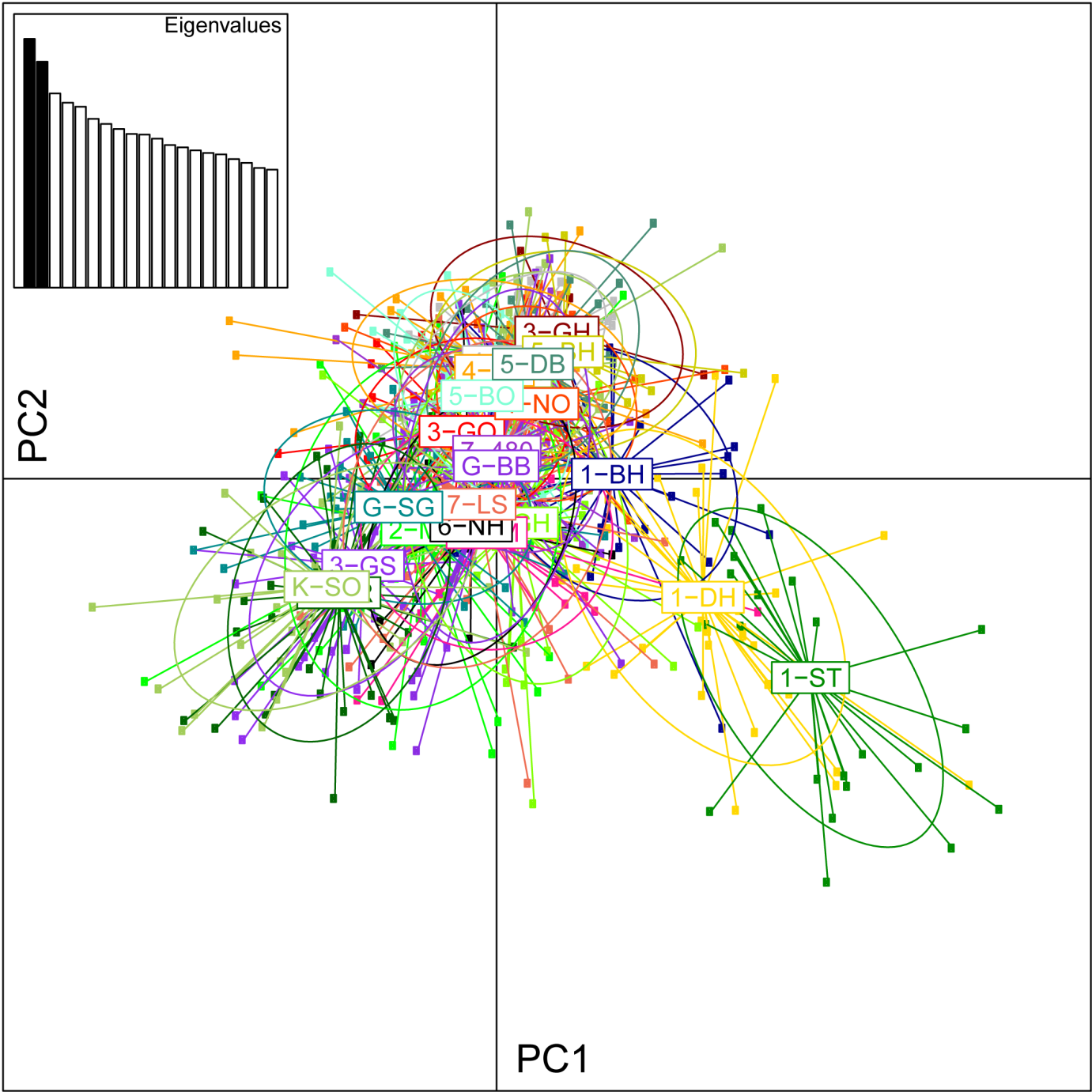


**Fig. S4** Evaluation of outliers. PCA analysis **without the 2 loci detected to be under positive selection** (C2 and G4, Fig. S2) in Lositan, the only method that identified loci under balancing selection. The two loci putatively under positive selection do not change the shape of the PCA based on all loci (shown in Fig. S2). Each dot represents an individual contained into populations by an ellipse of the population specific colour. Each ellipse represents 95 % of the inertia of the corresponding group. Shown in the insert box are the eigenvalues for the principal components (PCs), with the first two PCs shown in the PCA highlighted in black, which together explain 42% of the variance.

**
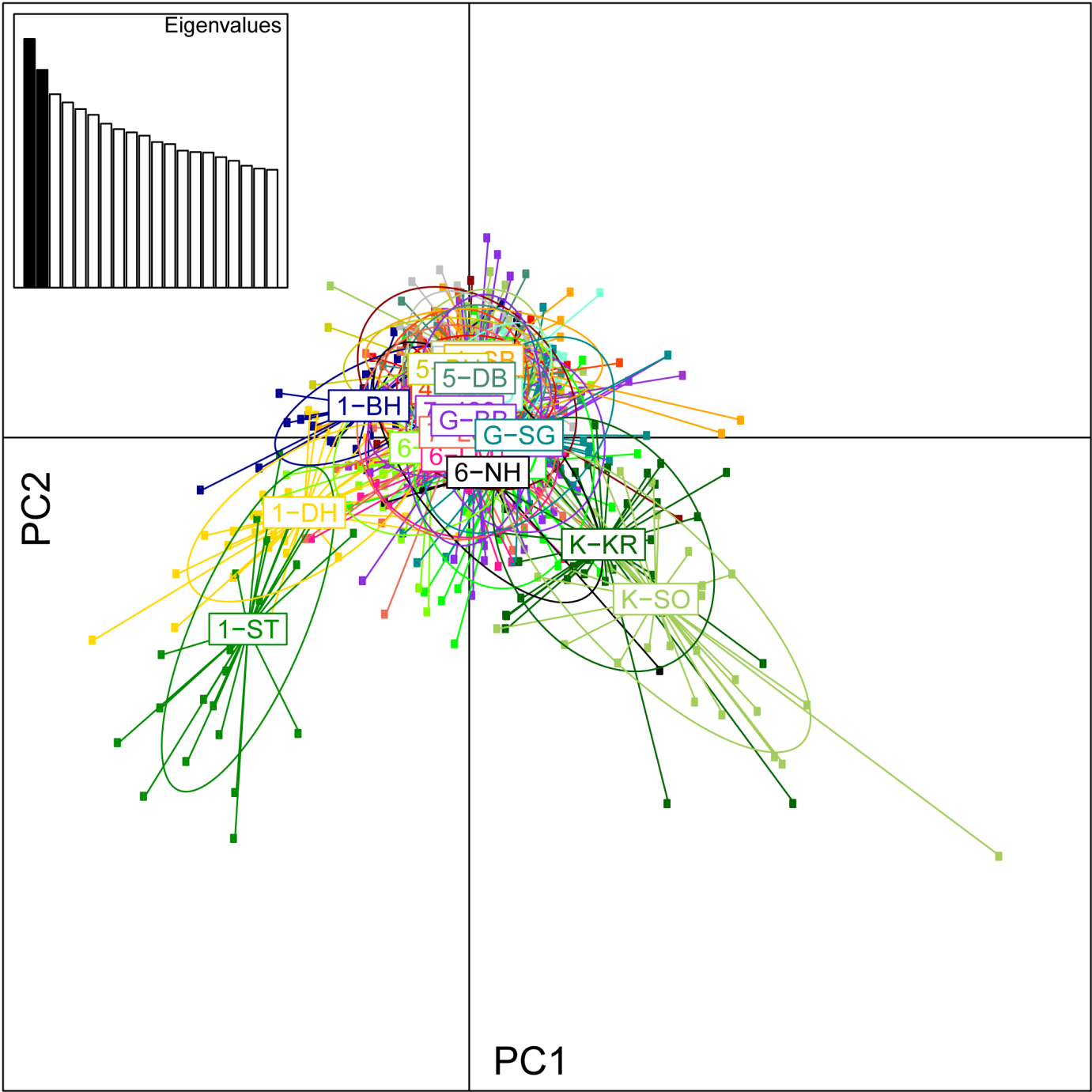
**

Fig. S5 Principal component analysis to test whether the studied region is a secondary contact zone between the Baltic and the North Sea. All sites assessed in this study are shown in blue. One location in the Baltic (FI-A and FI-B, Finland, 59.55 N, 21.47 E) is shown in red and one location from the North Eastern North Sea is shown in yellow (NN-A and NN-B, Northern Norway, 67.21 N, 15.00 W, unpublished data). These locations represent the geographically closest sites for which genotyping is available with the same set of microsatellites. The A and B sites at each location are only few km apart. The North Sea sites are clearly genetically divergent from the here assessed sites in the Kattegat-Skagerrak, while the Baltic sites cluster together with the Kattegat-Skagerrak and additionally do not possess any private alleles.


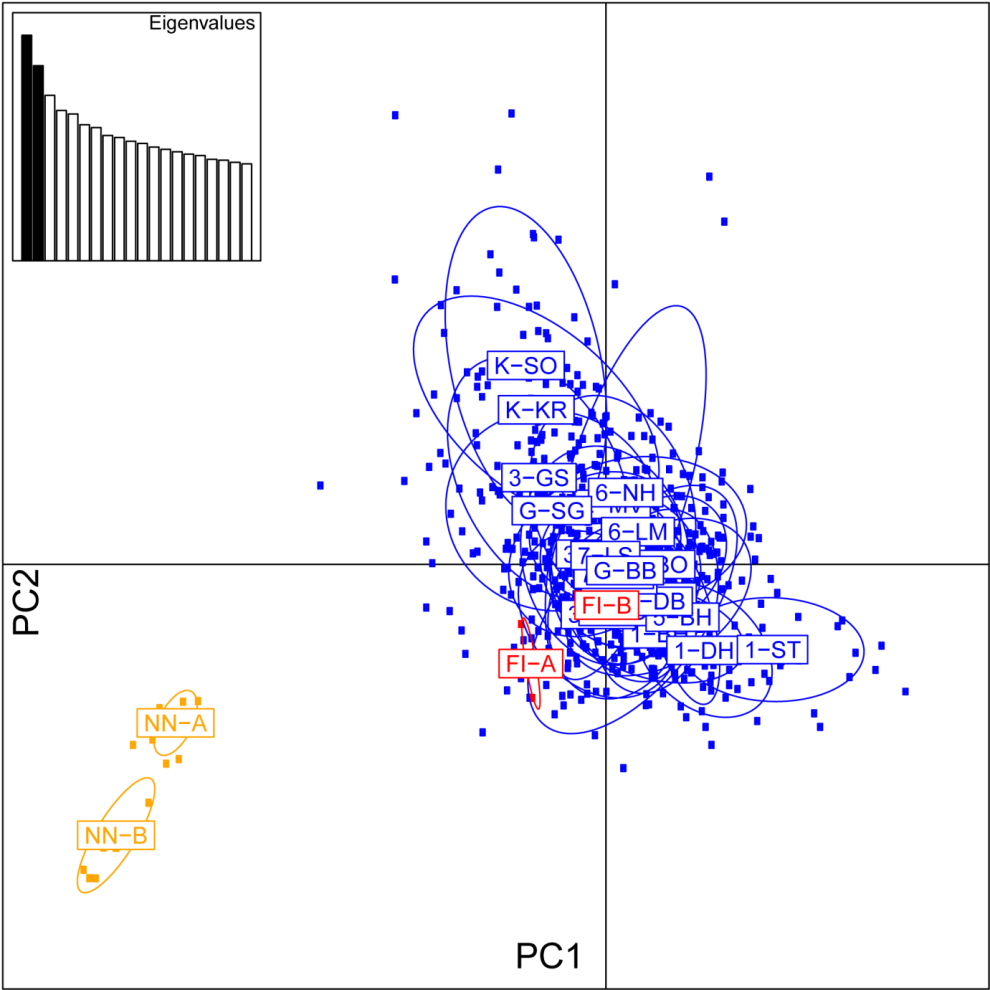


Table S3 POWSIM power simulation for the 20 microsatellite loci to evaluate the statistical power to detect population structure among sites. t is the simulated number of generations of drift used to obtain the desired *F*_ST._ Statistical power (shown as a probability assessed with Fisher’s method) is based on an effective population size of 2,000 with 100,000 batches and 1,000 replicates. Our 20 microsatellite loci have a 100% probability of being able to detect an *F*_ST_ as low as 0.0025.

| **20 loci** | | |
| --- | --- | --- |
| ***F*_ST_** | **t** | **power** |
| 0 | 0 | 0.067 |
| 0.00025 | 1 | 0.201 |
| 0.0025 | 10 | 1 |
| 0.025 | 100 | 1 |

**Table S4** Pairwise shared allele distance *D*_ps_ (1-ps) among the 23 *Zostera marina* meadows in the Skagerrak - Kattegat.

| pops | 1-BH | 1-ST | G-BB | 1-DH | 6-GH | 6-LM | 7-480 | 7-LS | 2-MV | 3-GS | 3-GO | 3-GH | 3-HH | 4-HO | 4-NO | K-SO | K-KR | G-SG | 4-SB | 5-BH | 5-DB | 5-BO | 6-NH |
| --- | --- | --- | --- | --- | --- | --- | --- | --- | --- | --- | --- | --- | --- | --- | --- | --- | --- | --- | --- | --- | --- | --- | --- |
| 1-BH | 0.00 | 0.24 | 0.22 | 0.19 | 0.18 | 0.20 | 0.21 | 0.19 | 0.22 | 0.25 | 0.20 | 0.25 | 0.22 | 0.18 | 0.19 | 0.26 | 0.27 | 0.25 | 0.19 | 0.23 | 0.19 | 0.21 | 0.22 |
| 1-ST | 0.24 | 0.00 | 0.26 | 0.19 | 0.26 | 0.27 | 0.29 | 0.27 | 0.30 | 0.33 | 0.30 | 0.27 | 0.26 | 0.27 | 0.24 | 0.36 | 0.35 | 0.32 | 0.27 | 0.28 | 0.26 | 0.31 | 0.29 |
| G-BB | 0.22 | 0.26 | 0.00 | 0.23 | 0.21 | 0.17 | 0.19 | 0.17 | 0.18 | 0.21 | 0.21 | 0.23 | 0.16 | 0.17 | 0.16 | 0.24 | 0.23 | 0.22 | 0.17 | 0.21 | 0.18 | 0.21 | 0.20 |
| 1-DH | 0.19 | 0.19 | 0.23 | 0.00 | 0.20 | 0.23 | 0.23 | 0.22 | 0.26 | 0.28 | 0.25 | 0.26 | 0.25 | 0.24 | 0.20 | 0.30 | 0.30 | 0.27 | 0.24 | 0.26 | 0.23 | 0.28 | 0.24 |
| 6-GH | 0.18 | 0.26 | 0.21 | 0.20 | 0.00 | 0.17 | 0.15 | 0.14 | 0.20 | 0.22 | 0.17 | 0.27 | 0.21 | 0.17 | 0.19 | 0.24 | 0.24 | 0.19 | 0.16 | 0.26 | 0.21 | 0.17 | 0.20 |
| 6-LM | 0.20 | 0.27 | 0.17 | 0.23 | 0.17 | 0.00 | 0.16 | 0.14 | 0.15 | 0.20 | 0.17 | 0.26 | 0.20 | 0.18 | 0.17 | 0.23 | 0.21 | 0.19 | 0.18 | 0.22 | 0.19 | 0.18 | 0.18 |
| 7-480 | 0.21 | 0.29 | 0.19 | 0.23 | 0.15 | 0.16 | 0.00 | 0.09 | 0.18 | 0.19 | 0.17 | 0.24 | 0.18 | 0.15 | 0.19 | 0.25 | 0.24 | 0.18 | 0.14 | 0.23 | 0.19 | 0.16 | 0.19 |
| 7-LS | 0.19 | 0.27 | 0.17 | 0.22 | 0.14 | 0.14 | 0.09 | 0.00 | 0.14 | 0.16 | 0.15 | 0.23 | 0.17 | 0.16 | 0.18 | 0.23 | 0.22 | 0.18 | 0.15 | 0.23 | 0.19 | 0.16 | 0.18 |
| 2-MV | 0.22 | 0.30 | 0.18 | 0.26 | 0.20 | 0.15 | 0.18 | 0.14 | 0.00 | 0.17 | 0.17 | 0.27 | 0.21 | 0.20 | 0.21 | 0.23 | 0.21 | 0.18 | 0.20 | 0.23 | 0.22 | 0.19 | 0.20 |
| 3-GS | 0.25 | 0.33 | 0.21 | 0.28 | 0.22 | 0.20 | 0.19 | 0.16 | 0.17 | 0.00 | 0.20 | 0.26 | 0.20 | 0.22 | 0.22 | 0.22 | 0.19 | 0.15 | 0.21 | 0.28 | 0.24 | 0.23 | 0.22 |
| 3-GO | 0.20 | 0.30 | 0.21 | 0.25 | 0.17 | 0.17 | 0.17 | 0.15 | 0.17 | 0.20 | 0.00 | 0.21 | 0.20 | 0.17 | 0.17 | 0.23 | 0.23 | 0.19 | 0.16 | 0.21 | 0.17 | 0.17 | 0.18 |
| 3-GH | 0.25 | 0.27 | 0.23 | 0.26 | 0.27 | 0.26 | 0.24 | 0.23 | 0.27 | 0.26 | 0.21 | 0.00 | 0.20 | 0.20 | 0.18 | 0.28 | 0.29 | 0.25 | 0.20 | 0.21 | 0.18 | 0.23 | 0.26 |
| 3-HH | 0.22 | 0.26 | 0.16 | 0.25 | 0.21 | 0.20 | 0.18 | 0.17 | 0.21 | 0.20 | 0.20 | 0.20 | 0.00 | 0.12 | 0.16 | 0.26 | 0.25 | 0.18 | 0.12 | 0.20 | 0.15 | 0.21 | 0.19 |
| 4-HO | 0.18 | 0.27 | 0.17 | 0.24 | 0.17 | 0.18 | 0.15 | 0.16 | 0.20 | 0.22 | 0.17 | 0.20 | 0.12 | 0.00 | 0.15 | 0.25 | 0.23 | 0.20 | 0.11 | 0.18 | 0.13 | 0.16 | 0.18 |
| 4-NO | 0.19 | 0.24 | 0.16 | 0.20 | 0.19 | 0.17 | 0.19 | 0.18 | 0.21 | 0.22 | 0.17 | 0.18 | 0.16 | 0.15 | 0.00 | 0.25 | 0.25 | 0.21 | 0.15 | 0.19 | 0.14 | 0.19 | 0.19 |
| K-SO | 0.26 | 0.36 | 0.24 | 0.30 | 0.24 | 0.23 | 0.25 | 0.23 | 0.23 | 0.22 | 0.23 | 0.28 | 0.26 | 0.25 | 0.25 | 0.00 | 0.15 | 0.22 | 0.24 | 0.31 | 0.25 | 0.26 | 0.22 |
| K-KR | 0.27 | 0.35 | 0.23 | 0.30 | 0.24 | 0.21 | 0.24 | 0.22 | 0.21 | 0.19 | 0.23 | 0.29 | 0.25 | 0.23 | 0.25 | 0.15 | 0.00 | 0.19 | 0.24 | 0.31 | 0.25 | 0.24 | 0.21 |
| G-SG | 0.25 | 0.32 | 0.22 | 0.27 | 0.19 | 0.19 | 0.18 | 0.18 | 0.18 | 0.15 | 0.19 | 0.25 | 0.18 | 0.20 | 0.21 | 0.22 | 0.19 | 0.00 | 0.18 | 0.26 | 0.22 | 0.21 | 0.21 |
| 4-SB | 0.19 | 0.27 | 0.17 | 0.24 | 0.16 | 0.18 | 0.14 | 0.15 | 0.20 | 0.21 | 0.16 | 0.20 | 0.12 | 0.11 | 0.15 | 0.24 | 0.24 | 0.18 | 0.00 | 0.21 | 0.15 | 0.17 | 0.19 |
| 5-BH | 0.23 | 0.28 | 0.21 | 0.26 | 0.26 | 0.22 | 0.23 | 0.23 | 0.23 | 0.28 | 0.21 | 0.21 | 0.20 | 0.18 | 0.19 | 0.31 | 0.31 | 0.26 | 0.21 | 0.00 | 0.16 | 0.21 | 0.24 |
| 5-DB | 0.19 | 0.26 | 0.18 | 0.23 | 0.21 | 0.19 | 0.19 | 0.19 | 0.22 | 0.24 | 0.17 | 0.18 | 0.15 | 0.13 | 0.14 | 0.25 | 0.25 | 0.22 | 0.15 | 0.16 | 0.00 | 0.18 | 0.21 |
| 5-BO | 0.21 | 0.31 | 0.21 | 0.28 | 0.17 | 0.18 | 0.16 | 0.16 | 0.19 | 0.23 | 0.17 | 0.23 | 0.21 | 0.16 | 0.19 | 0.26 | 0.24 | 0.21 | 0.17 | 0.21 | 0.18 | 0.00 | 0.20 |
| 6-NH | 0.22 | 0.29 | 0.20 | 0.24 | 0.20 | 0.18 | 0.19 | 0.18 | 0.20 | 0.22 | 0.18 | 0.26 | 0.19 | 0.18 | 0.19 | 0.22 | 0.21 | 0.21 | 0.19 | 0.24 | 0.21 | 0.20 | 0.00 |

**Table S5** Pairwise Weir & Cockerham *F*_ST_ among populations calculated with Arlequin 3.5 ([Excoffier & Lischer 2010](#_ENREF_4)). All pairwise estimates are significant (p<0.05) and actual values are shown above the diagonal. After Bonferroni corrections, seven population pairs (out of 253) show non significant p-values: The geographically close sites 7-LS and 6-LM, 7-LS and 7-480, 6-NH and 6-LM, 7-LS and 2-MV, 6-NH and 7-LS, 4-HO and 4-SB and K-SO and K-KR. *F*_ST_ measures deviation from panmixia and is calculated based on allele frequencies. Values range in theory from zero to one, but *F*_ST_ does not account for the fact that the maximum attainable *F*_ST_ is dependent on heterozygosity.

| pops | 1-BH | 1-  ST | G-BB | 1-DH | 6-GH | 6-LM | 7-480 | 7-LS | 2-MV | 3-GS | 3-GO | 3-GH | 3-HH | 4-  HO | 4-NO | K-SO | K-KR | G-SG | 4-SB | 5-BH | 5-DB | 5-BO | 6-  NH |
| --- | --- | --- | --- | --- | --- | --- | --- | --- | --- | --- | --- | --- | --- | --- | --- | --- | --- | --- | --- | --- | --- | --- | --- |
| 1-BH | na | 0+-0 | 0+-0 | 0+-0 | 0+-0 | 0+-0 | 0+-0 | 0+-0 | 0+-0 | 0+-0 | 0+-0 | 0+-0 | 0+-0 | 0+-0 | 0+-0 | 0+-0 | 0+-0 | 0+-0 | 0+-0 | 0+-0 | 0+-0 | 0+-0 | 0+-0 |
| 1-ST | 0.14 | na | 0+-0 | 0+-0 | 0+-0 | 0+-0 | 0+-0 | 0+-0 | 0+-0 | 0+-0 | 0+-0 | 0+-0 | 0+-0 | 0+-0 | 0+-0 | 0+-0 | 0+-0 | 0+-0 | 0+-0 | 0+-0 | 0+-0 | 0+-0 | 0+-0 |
| G-BB | 0.10 | 0.16 | na | 0+-0 | 0+-0 | 0+-0 | 0+-0 | 0+-0 | 0+-0 | 0+-0 | 0+-0 | 0+-0 | 0+-0 | 0+-0 | 0+-0 | 0+-0 | 0+-0 | 0+-0 | 0+-0 | 0+-0 | 0+-0 | 0+-0 | 0+-0 |
| 1-DH | 0.07 | 0.08 | 0.10 | na | 0+-0 | 0+-0 | 0+-0 | 0+-0 | 0+-0 | 0+-0 | 0+-0 | 0+-0 | 0+-0 | 0+-0 | 0+-0 | 0+-0 | 0+-0 | 0+-0 | 0+-0 | 0+-0 | 0+-0 | 0+-0 | 0+-0 |
| 6-GH | 0.08 | 0.16 | 0.08 | 0.08 | na | 0+-0 | 0+-0 | 0+-0 | 0+-0 | 0+-0 | 0+-0 | 0+-0 | 0+-0 | 0+-0 | 0+-0 | 0+-0 | 0+-0 | 0+-0 | 0+-0 | 0+-0 | 0+-0 | 0+-0 | 0+-0 |
| 6-LM | 0.06 | 0.13 | 0.04 | 0.08 | 0.04 | na | 0+-0 | 0.00099+-0.0003 | 0+-0 | 0+-0 | 0+-0 | 0+-0 | 0+-0 | 0+-0 | 0+-0 | 0+-0 | 0+-0 | 0+-0 | 0+-0 | 0+-0 | 0+-0 | 0+-0 | 0.00158+-0.0004 |
| 7-480 | 0.11 | 0.18 | 0.07 | 0.11 | 0.04 | 0.04 | na | 0.04287+-0.0021 | 0+-0 | 0+-0 | 0+-0 | 0+-0 | 0+-0 | 0+-0 | 0+-0 | 0+-0 | 0+-0 | 0+-0 | 0+-0 | 0+-0 | 0+-0 | 0+-0 | 0+-0 |
| 7-LS | 0.07 | 0.17 | 0.05 | 0.09 | 0.02 | 0.02 | 0.01 | na | 0.00020+-0.0001 | 0+-0 | 0+-0 | 0+-0 | 0+-0 | 0+-0 | 0+-0 | 0+-0 | 0+-0 | 0+-0 | 0+-0 | 0+-0 | 0+-0 | 0+-0 | 0.00030+-0.0002 |
| 2-MV | 0.10 | 0.18 | 0.05 | 0.12 | 0.07 | 0.02 | 0.06 | 0.02 | na | 0+-0 | 0+-0 | 0+-0 | 0+-0 | 0+-0 | 0+-0 | 0+-0 | 0+-0 | 0+-0 | 0+-0 | 0+-0 | 0+-0 | 0+-0 | 0+-0 |
| 3-GS | 0.12 | 0.21 | 0.08 | 0.14 | 0.08 | 0.05 | 0.08 | 0.04 | 0.04 | na | 0+-0 | 0+-0 | 0+-0 | 0+-0 | 0+-0 | 0+-0 | 0+-0 | 0+-0 | 0+-0 | 0+-0 | 0+-0 | 0+-0 | 0+-0 |
| 3-GO | 0.08 | 0.19 | 0.08 | 0.12 | 0.06 | 0.03 | 0.06 | 0.03 | 0.04 | 0.06 | na | 0+-0 | 0+-0 | 0+-0 | 0+-0 | 0+-0 | 0+-0 | 0+-0 | 0+-0 | 0+-0 | 0+-0 | 0+-0 | 0+-0 |
| 3-GH | 0.17 | 0.20 | 0.11 | 0.15 | 0.16 | 0.11 | 0.11 | 0.11 | 0.12 | 0.15 | 0.10 | na | 0+-0 | 0+-0 | 0+-0 | 0+-0 | 0+-0 | 0+-0 | 0+-0 | 0+-0 | 0+-0 | 0+-0 | 0+-0 |
| 3-HH | 0.12 | 0.19 | 0.04 | 0.13 | 0.09 | 0.07 | 0.07 | 0.06 | 0.09 | 0.09 | 0.09 | 0.09 | na | 0+-0 | 0+-0 | 0+-0 | 0+-0 | 0+-0 | 0+-0 | 0+-0 | 0+-0 | 0+-0 | 0+-0 |
| 4-HO | 0.09 | 0.18 | 0.05 | 0.12 | 0.06 | 0.05 | 0.04 | 0.04 | 0.07 | 0.11 | 0.05 | 0.09 | 0.02 | na | 0+-0 | 0+-0 | 0+-0 | 0+-0 | 0.00634+-0.0008 | 0+-0 | 0+-0 | 0+-0 | 0+-0 |
| 4-NO | 0.07 | 0.14 | 0.04 | 0.07 | 0.07 | 0.04 | 0.07 | 0.06 | 0.07 | 0.09 | 0.06 | 0.08 | 0.05 | 0.04 | na | 0+-0 | 0+-0 | 0+-0 | 0+-0 | 0+-0 | 0+-0 | 0+-0 | 0+-0 |
| K-SO | 0.14 | 0.21 | 0.09 | 0.15 | 0.11 | 0.07 | 0.12 | 0.09 | 0.07 | 0.07 | 0.09 | 0.15 | 0.12 | 0.12 | 0.11 | na | 0.00248+-0.0005 | 0+-0 | 0+-0 | 0+-0 | 0+-0 | 0+-0 | 0+-0 |
| K-KR | 0.13 | 0.20 | 0.08 | 0.15 | 0.10 | 0.05 | 0.12 | 0.08 | 0.06 | 0.05 | 0.09 | 0.17 | 0.12 | 0.12 | 0.11 | 0.02 | na | 0+-0 | 0+-0 | 0+-0 | 0+-0 | 0+-0 | 0+-0 |
| G-SG | 0.13 | 0.20 | 0.09 | 0.15 | 0.06 | 0.05 | 0.06 | 0.04 | 0.05 | 0.03 | 0.06 | 0.14 | 0.09 | 0.08 | 0.10 | 0.08 | 0.06 | na | 0+-0 | 0+-0 | 0+-0 | 0+-0 | 0+-0 |
| 4-SB | 0.09 | 0.17 | 0.05 | 0.10 | 0.05 | 0.04 | 0.03 | 0.03 | 0.06 | 0.08 | 0.05 | 0.08 | 0.02 | 0.01 | 0.04 | 0.11 | 0.11 | 0.07 | na | 0+-0 | 0+-0 | 0+-0 | 0+-0 |
| 5-BH | 0.12 | 0.17 | 0.08 | 0.14 | 0.14 | 0.08 | 0.10 | 0.10 | 0.09 | 0.15 | 0.09 | 0.09 | 0.08 | 0.06 | 0.07 | 0.16 | 0.17 | 0.14 | 0.08 | na | 0+-0 | 0+-0 | 0+-0 |
| 5-DB | 0.09 | 0.16 | 0.06 | 0.10 | 0.08 | 0.06 | 0.08 | 0.07 | 0.09 | 0.12 | 0.06 | 0.07 | 0.04 | 0.03 | 0.03 | 0.11 | 0.12 | 0.11 | 0.03 | 0.04 | na | 0+-0 | 0+-0 |
| 5-BO | 0.13 | 0.21 | 0.09 | 0.16 | 0.08 | 0.06 | 0.05 | 0.06 | 0.06 | 0.12 | 0.07 | 0.14 | 0.11 | 0.06 | 0.08 | 0.14 | 0.13 | 0.09 | 0.07 | 0.09 | 0.08 | na | 0+-0 |
| 6-NH | 0.09 | 0.14 | 0.05 | 0.09 | 0.06 | 0.03 | 0.05 | 0.03 | 0.04 | 0.06 | 0.04 | 0.12 | 0.06 | 0.05 | 0.05 | 0.07 | 0.06 | 0.06 | 0.05 | 0.10 | 0.07 | 0.08 | na |

**Table S6** Pairwise *G’*_ST_ ([Hedrick 2005](#_ENREF_7)) among populations was calculated with the diveRsity package ([Keenan *et al.* 2013](#_ENREF_9)) in R 3.2.2 using 1,000 bootstrap replicates to test for the significance of pairwise comparisons using confidence intervals. **Non**-significant pairwise genetic differentiation estimates are shown in bold. *G’*_ST_ takes into account the maximum heterozygosity possible to ensure that *G’*_ST_ can range from zero to one.

| pops | 1-BH | 1-ST | G-BB | 1-DH | 6-GH | 6-LM | 7-480 | 7-LS | 2-MV | 3-GS | 3-GO | 3-GH | 3-HH | 4-HO | 4-NO | K-SO | K-KR | G-SG | 4-SB | 5-BH | 5-DB | 5-BO | 6-NH |
| --- | --- | --- | --- | --- | --- | --- | --- | --- | --- | --- | --- | --- | --- | --- | --- | --- | --- | --- | --- | --- | --- | --- | --- |
| 1-BH |  |  |  |  |  |  |  |  |  |  |  |  |  |  |  |  |  |  |  |  |  |  |  |
| 1-ST | 0.22 |  |  |  |  |  |  |  |  |  |  |  |  |  |  |  |  |  |  |  |  |  |  |
| G-BB | 0.16 | 0.25 |  |  |  |  |  |  |  |  |  |  |  |  |  |  |  |  |  |  |  |  |  |
| 1-DH | 0.11 | 0.13 | 0.17 |  |  |  |  |  |  |  |  |  |  |  |  |  |  |  |  |  |  |  |  |
| 6-GH | 0.12 | 0.25 | 0.13 | 0.13 |  |  |  |  |  |  |  |  |  |  |  |  |  |  |  |  |  |  |  |
| 6-LM | 0.09 | 0.22 | 0.07 | 0.14 | 0.07 |  |  |  |  |  |  |  |  |  |  |  |  |  |  |  |  |  |  |
| 7-480 | 0.16 | 0.29 | 0.12 | 0.18 | 0.06 | 0.07 |  |  |  |  |  |  |  |  |  |  |  |  |  |  |  |  |  |
| 7-LS | 0.12 | 0.27 | 0.09 | 0.15 | 0.04 | **0.03** | **0.01** |  |  |  |  |  |  |  |  |  |  |  |  |  |  |  |  |
| 2-MV | 0.16 | 0.31 | 0.09 | 0.20 | 0.12 | 0.04 | 0.10 | 0.04 |  |  |  |  |  |  |  |  |  |  |  |  |  |  |  |
| 3-GS | 0.20 | 0.36 | 0.13 | 0.23 | 0.13 | 0.08 | 0.14 | 0.07 | 0.06 |  |  |  |  |  |  |  |  |  |  |  |  |  |  |
| 3-GO | 0.12 | 0.30 | 0.13 | 0.19 | 0.10 | 0.05 | 0.09 | 0.05 | 0.07 | 0.10 |  |  |  |  |  |  |  |  |  |  |  |  |  |
| 3-GH | 0.25 | 0.32 | 0.18 | 0.23 | 0.25 | 0.19 | 0.18 | 0.17 | 0.21 | 0.25 | 0.16 |  |  |  |  |  |  |  |  |  |  |  |  |
| 3-HH | 0.18 | 0.29 | 0.07 | 0.21 | 0.14 | 0.11 | 0.11 | 0.10 | 0.14 | 0.15 | 0.13 | 0.14 |  |  |  |  |  |  |  |  |  |  |  |
| 4-HO | 0.13 | 0.28 | 0.09 | 0.18 | 0.09 | 0.07 | 0.07 | 0.07 | 0.12 | 0.17 | 0.09 | 0.14 | 0.04 |  |  |  |  |  |  |  |  |  |  |
| 4-NO | 0.10 | 0.22 | 0.06 | 0.12 | 0.11 | 0.07 | 0.12 | 0.10 | 0.11 | 0.15 | 0.10 | 0.12 | 0.07 | 0.06 |  |  |  |  |  |  |  |  |  |
| K-SO | 0.23 | 0.35 | 0.14 | 0.24 | 0.18 | 0.13 | 0.20 | 0.15 | 0.13 | 0.13 | 0.15 | 0.25 | 0.19 | 0.20 | 0.18 |  |  |  |  |  |  |  |  |
| K-KR | 0.21 | 0.34 | 0.13 | 0.25 | 0.17 | 0.09 | 0.20 | 0.14 | 0.11 | 0.09 | 0.15 | 0.30 | 0.19 | 0.20 | 0.18 | **0.03** |  |  |  |  |  |  |  |
| G-SG | 0.21 | 0.34 | 0.15 | 0.24 | 0.10 | 0.08 | 0.10 | 0.07 | 0.08 | 0.05 | 0.10 | 0.23 | 0.14 | 0.13 | 0.16 | 0.14 | 0.10 |  |  |  |  |  |  |
| 4-SB | 0.13 | 0.27 | 0.08 | 0.16 | 0.08 | 0.07 | 0.05 | 0.05 | 0.10 | 0.14 | 0.08 | 0.13 | 0.03 | **0.02** | 0.06 | 0.18 | 0.18 | 0.11 |  |  |  |  |  |
| 5-BH | 0.17 | 0.28 | 0.13 | 0.23 | 0.22 | 0.14 | 0.16 | 0.16 | 0.16 | 0.25 | 0.14 | 0.14 | 0.13 | 0.09 | 0.11 | 0.27 | 0.29 | 0.24 | 0.12 |  |  |  |  |
| 5-DB | 0.14 | 0.25 | 0.09 | 0.15 | 0.13 | 0.10 | 0.12 | 0.11 | 0.15 | 0.20 | 0.09 | 0.11 | 0.06 | 0.04 | 0.05 | 0.19 | 0.21 | 0.19 | 0.05 | 0.07 |  |  |  |
| 5-BO | 0.20 | 0.32 | 0.14 | 0.25 | 0.12 | **0.09** | 0.07 | 0.09 | 0.10 | 0.20 | 0.10 | 0.20 | 0.16 | 0.09 | 0.13 | 0.23 | 0.22 | 0.15 | 0.10 | 0.14 | 0.12 |  |  |
| 6-NH | 0.13 | 0.24 | 0.08 | 0.15 | 0.09 | 0.05 | 0.08 | 0.05 | 0.07 | 0.11 | 0.07 | 0.19 | 0.10 | 0.08 | 0.08 | 0.12 | 0.10 | 0.11 | 0.08 | 0.17 | 0.11 | 0.12 |  |

**Table S7** Pairwise Jost’s *D*_EST_ ([Jost 2008](#_ENREF_8)) among populations was calculated with the diveRsity package ([Keenan *et al.* 2013](#_ENREF_9)) in R 3.2.2 using 1,000 bootstrap replicates to test for the significance of pairwise comparisons using confidence intervals. **Non**- significant pairwise genetic differentiation estimates are shown in bold. *D*_EST_ measures deviation from complete differentiation and is based on the effective number of alleles.

| pops | 1-BH | 1-ST | G-BB | 1-DH | 6-GH | 6-LM | 7-480 | 7-LS | 2-MV | 3-GS | 3-GO | 3-GH | 3-HH | 4-HO | 4-NO | K-SO | K-KR | G-SG | 4-SB | 5-BH | 5-DB | 5-BO | 6-NH |
| --- | --- | --- | --- | --- | --- | --- | --- | --- | --- | --- | --- | --- | --- | --- | --- | --- | --- | --- | --- | --- | --- | --- | --- |
| 1-BH |  |  |  |  |  |  |  |  |  |  |  |  |  |  |  |  |  |  |  |  |  |  |  |
| 1-ST | 0.03 |  |  |  |  |  |  |  |  |  |  |  |  |  |  |  |  |  |  |  |  |  |  |
| G-BB | 0.03 | 0.04 |  |  |  |  |  |  |  |  |  |  |  |  |  |  |  |  |  |  |  |  |  |
| 1-DH | 0.02 | 0.02 | 0.03 |  |  |  |  |  |  |  |  |  |  |  |  |  |  |  |  |  |  |  |  |
| 6-GH | 0.02 | 0.05 | 0.03 | 0.02 |  |  |  |  |  |  |  |  |  |  |  |  |  |  |  |  |  |  |  |
| 6-LM | 0.02 | 0.05 | 0.01 | 0.03 | 0.01 |  |  |  |  |  |  |  |  |  |  |  |  |  |  |  |  |  |  |
| 7-480 | 0.02 | 0.05 | 0.02 | 0.04 | 0.01 | **0.01** |  |  |  |  |  |  |  |  |  |  |  |  |  |  |  |  |  |
| 7-LS | 0.01 | 0.04 | 0.01 | 0.03 | 0.01 | **0.00** | **0.00** |  |  |  |  |  |  |  |  |  |  |  |  |  |  |  |  |
| 2-MV | 0.03 | 0.06 | 0.01 | 0.04 | 0.02 | **0.01** | 0.01 | 0.01 |  |  |  |  |  |  |  |  |  |  |  |  |  |  |  |
| 3-GS | 0.04 | 0.08 | 0.02 | 0.06 | 0.03 | 0.02 | 0.01 | 0.01 | 0.01 |  |  |  |  |  |  |  |  |  |  |  |  |  |  |
| 3-GO | 0.02 | 0.05 | 0.02 | 0.04 | 0.01 | 0.01 | 0.01 | **0.01** | 0.01 | 0.02 |  |  |  |  |  |  |  |  |  |  |  |  |  |
| 3-GH | 0.04 | 0.03 | 0.03 | 0.04 | 0.06 | 0.04 | 0.04 | 0.03 | 0.04 | 0.04 | 0.02 |  |  |  |  |  |  |  |  |  |  |  |  |
| 3-HH | 0.03 | 0.04 | 0.01 | 0.04 | 0.02 | 0.01 | 0.01 | 0.01 | 0.02 | 0.02 | 0.02 | 0.02 |  |  |  |  |  |  |  |  |  |  |  |
| 4-HO | 0.02 | 0.04 | 0.01 | 0.03 | 0.02 | 0.01 | 0.01 | 0.01 | 0.02 | 0.03 | 0.02 | 0.02 | 0.01 |  |  |  |  |  |  |  |  |  |  |
| 4-NO | 0.02 | 0.03 | 0.01 | 0.02 | 0.02 | 0.01 | 0.02 | 0.01 | 0.02 | 0.03 | 0.01 | 0.02 | 0.01 | 0.01 |  |  |  |  |  |  |  |  |  |
| K-SO | 0.05 | 0.10 | 0.04 | 0.07 | 0.04 | 0.04 | 0.04 | 0.04 | 0.03 | 0.03 | 0.04 | 0.05 | 0.04 | 0.04 | 0.04 |  |  |  |  |  |  |  |  |
| K-KR | 0.05 | 0.11 | 0.03 | 0.07 | 0.03 | 0.02 | 0.03 | 0.03 | 0.02 | 0.01 | 0.03 | 0.05 | 0.04 | 0.03 | 0.04 | **0.01** |  |  |  |  |  |  |  |
| G-SG | 0.05 | 0.07 | 0.02 | 0.06 | 0.02 | 0.02 | 0.02 | 0.02 | 0.01 | **0.01** | 0.02 | 0.04 | 0.01 | 0.02 | 0.03 | 0.03 | 0.02 |  |  |  |  |  |  |
| 4-SB | 0.02 | 0.04 | 0.01 | 0.03 | 0.02 | 0.02 | 0.01 | 0.01 | 0.02 | 0.02 | 0.01 | 0.02 | **0.00** | **0.00** | 0.01 | 0.04 | 0.04 | 0.02 |  |  |  |  |  |
| 5-BH | 0.02 | 0.04 | 0.02 | 0.04 | 0.04 | 0.02 | 0.03 | 0.03 | 0.03 | 0.04 | 0.02 | 0.02 | 0.02 | 0.01 | 0.02 | 0.07 | 0.05 | 0.05 | 0.02 |  |  |  |  |
| 5-DB | 0.02 | 0.03 | 0.01 | 0.03 | 0.02 | 0.01 | 0.02 | 0.01 | 0.02 | 0.03 | 0.01 | **0.01** | 0.01 | 0.01 | 0.01 | 0.04 | 0.04 | 0.03 | 0.01 | 0.01 |  |  |  |
| 5-BO | 0.02 | 0.07 | 0.02 | 0.05 | 0.01 | 0.01 | 0.01 | 0.01 | 0.01 | 0.02 | 0.01 | 0.03 | 0.02 | 0.01 | 0.02 | 0.05 | 0.03 | 0.03 | 0.01 | 0.02 | 0.01 |  |  |
| 6-NH | 0.03 | 0.04 | 0.02 | 0.03 | 0.02 | **0.01** | 0.01 | 0.01 | 0.01 | 0.02 | **0.01** | 0.04 | 0.02 | 0.01 | 0.01 | 0.02 | 0.01 | 0.02 | 0.01 | 0.03 | 0.02 | 0.02 |  |

**Fig. S6** The deviance information criterion (DIC) analysis for the TESS analysis (a) and deltaK for the Structure analysis (b) indicate the presence of 3 clusters (TESS) or 2 clusters (Structure) as the most likely population subdivision. DIC helps to determine if improvement in model fit justifies the use of a more complex model whereby model complexity is related to the number of parameters. The *ad hoc* statistic deltaK is based on the rate of change in the log probability of data between successive K values. The best model is identified by the lowest DIC (TESS) and the highest deltaK (Structure).

**
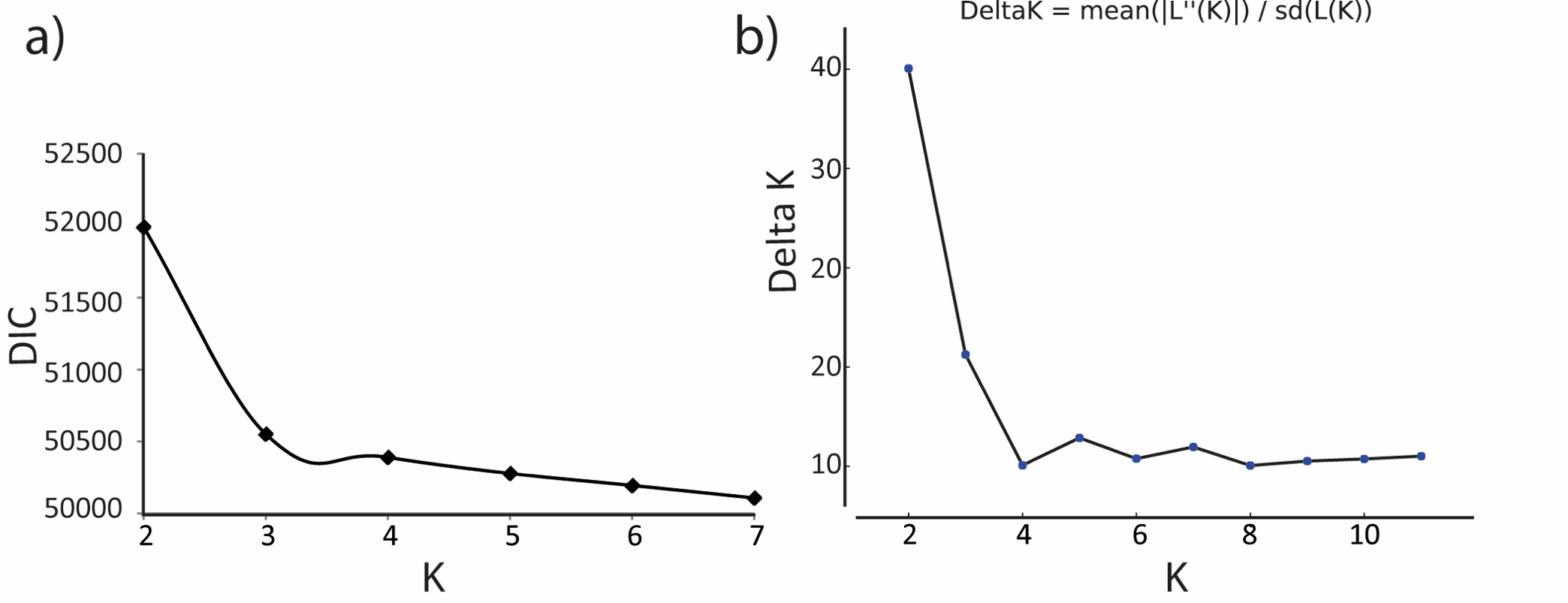
**

**Fig. S7** Comparison of the two clustering analyses performed by TESS and Structure.

For Structure, the scenario of K = 2 clusters was identified as the most likely, while TESS, which incorporates isolation by distance (IBD), suggested K_max_= 3 as the most likely scenario. Note that the colouring of the clusters for K = 3 is not identical to what is shown in Fig. 3, i.e. the giant blue cluster is shown here in light blue, and the green cluster of Northern sites is shown in dark blue. This was done to improve the visualization of the gradient of each cluster in Fig. 3.

Structure analyses were conducted using the admixture model with correlated allele frequencies, and sampling sites as prior (LOCPRIOR). The admixture model and the correlated allele frequencies between populations options were chosen as they perform best ([Falush *et al.* 2003](#_ENREF_5)). MCMC chains used a burn-in of 100,000 chains followed by 1,000,000 MCMC replications. Five independent chains were run for each K value from 1 to 25. We evaluated the most likely value of K using the method of Evanno *et al.* (2005) as implemented in STRUCTURE HARVESTER ([Earl & von Holdt 2012](#_ENREF_2)). Clumpak ([Kopelman *et al.* 2015](#_ENREF_11)) was used for post-processing of Structure results. See the main manuscript for details on the TESS analysis. TESS is generally better at capturing gradual change and takes into account geographic location. Both programs deliver extremely similar results for the scenario K = 3. No additional clustering is visible at Ks > 5 (not shown).


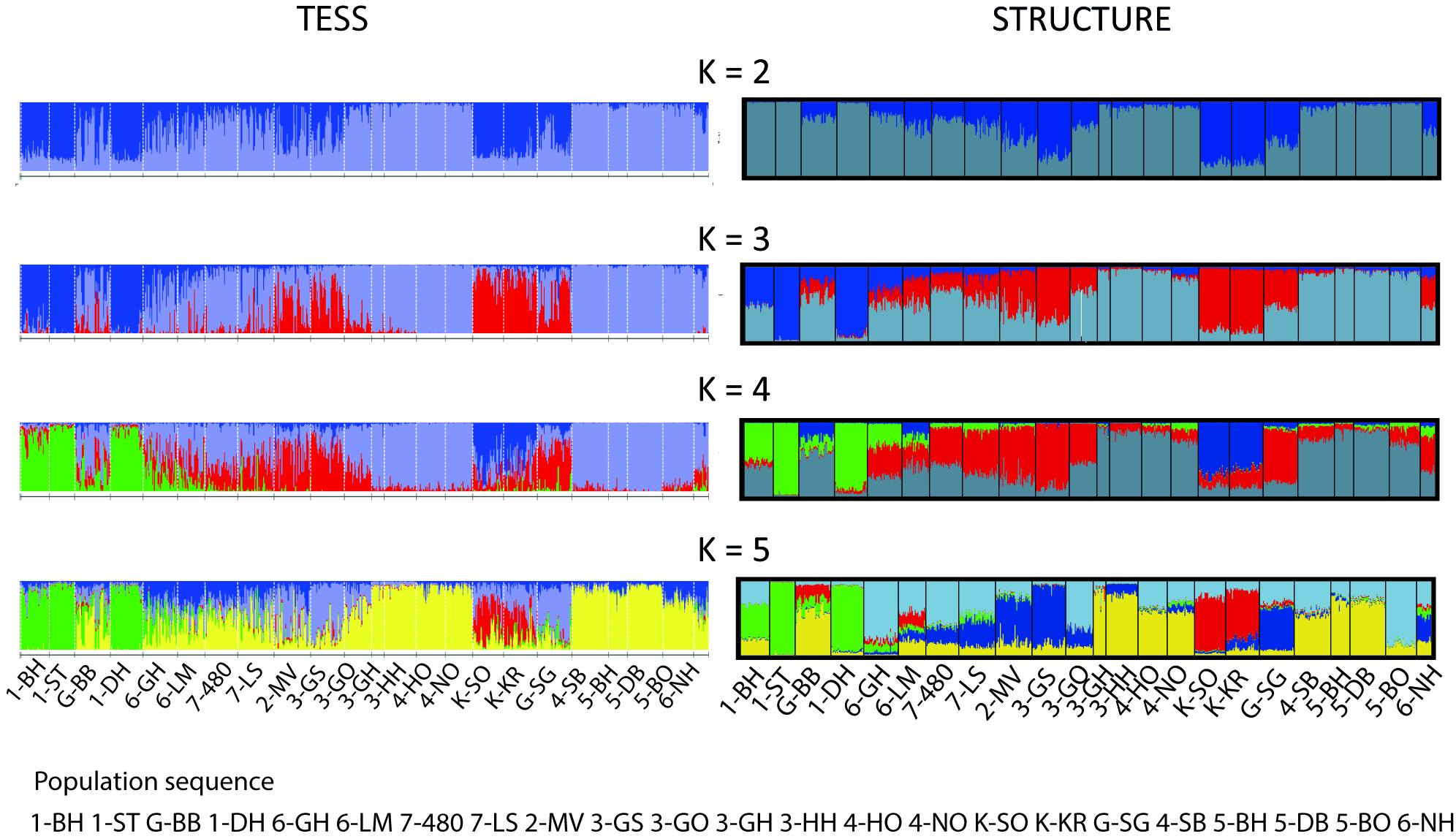


**Table S8** Asymmetric migration rates based on *G*_ST_ calculated with divMigrate online using an alpha of 0.05 and 1,000 bootstraps ([Sundqvist *et al.* 2016](#_ENREF_16)). Source populations are shown in columns, receiving populations in rows. Numbers in bold indicate statistically significant values based on the bootstraping.

|  | 1-BH | 1-ST | G-BB | 1-DH | 6-GH | 6-LM | 7-480 | 7-LS | 2-MV | 3-GS | 3-GO | 3-GH | 3-HH | 4-HO | 4-NO | K-SO | K-KR | G-SG | 4-SB | 5-BH | 5-DB | 5-BO | 6-NH |
| --- | --- | --- | --- | --- | --- | --- | --- | --- | --- | --- | --- | --- | --- | --- | --- | --- | --- | --- | --- | --- | --- | --- | --- |
| 1-BH | 0 | 0.063 | 0.122 | 0.121 | 0.134 | 0.136 | 0.144 | 0.178 | 0.098 | 0.075 | 0.12 | 0.057 | 0.116 | 0.151 | 0.13 | 0.054 | 0.063 | 0.074 | 0.144 | 0.113 | 0.128 | 0.096 | 0.098 |
| 1-ST | **0.166** | 0 | 0.081 | 0.145 | 0.077 | 0.081 | 0.078 | 0.088 | 0.073 | 0.065 | **0.065** | **0.094** | **0.102** | **0.1** | 0.091 | 0.05 | 0.053 | 0.059 | 0.097 | **0.077** | 0.096 | 0.049 | 0.064 |
| G-BB | 0.126 | 0.069 | 0 | 0.113 | 0.171 | 0.252 | 0.176 | 0.224 | 0.178 | 0.133 | 0.147 | 0.099 | **0.307** | 0.26 | 0.267 | 0.092 | 0.124 | 0.121 | 0.302 | 0.137 | 0.225 | 0.15 | 0.113 |
| 1-DH | **0.226** | 0.108 | 0.088 | 0 | 0.165 | 0.109 | 0.112 | 0.128 | 0.094 | 0.072 | 0.07 | 0.07 | **0.101** | **0.129** | 0.147 | 0.05 | 0.065 | 0.076 | 0.13 | **0.089** | 0.132 | 0.065 | 0.071 |
| 6-GH | 0.135 | 0.06 | 0.143 | 0.102 | 0 | 0.194 | 0.338 | 0.365 | 0.16 | 0.145 | 0.194 | 0.062 | 0.149 | 0.223 | 0.178 | 0.087 | 0.111 | 0.168 | 0.226 | 0.089 | 0.159 | 0.165 | 0.132 |
| 6-LM | 0.261 | 0.087 | 0.241 | 0.134 | **0.409** | 0 | 0.404 | 0.615 | 0.363 | 0.202 | 0.274 | 0.099 | 0.175 | 0.347 | 0.27 | 0.136 | 0.193 | 0.202 | 0.296 | **0.195** | 0.229 | **0.304** | 0.179 |
| 7-480 | 0.122 | 0.073 | 0.187 | 0.095 | 0.3 | 0.252 | 0 | 0.78 | 0.231 | 0.158 | 0.212 | 0.103 | 0.215 | 0.355 | 0.188 | 0.079 | 0.107 | 0.209 | 0.344 | 0.154 | 0.197 | 0.29 | 0.183 |
| 7-LS | 0.176 | 0.08 | 0.24 | 0.136 | 0.46 | 0.359 | 1 | 0 | 0.439 | 0.326 | 0.311 | 0.118 | 0.293 | 0.401 | 0.235 | 0.097 | 0.138 | 0.304 | 0.351 | **0.184** | 0.213 | 0.273 | 0.214 |
| 2-MV | 0.174 | 0.061 | 0.264 | 0.11 | 0.233 | 0.361 | 0.303 | 0.641 | 0 | 0.329 | 0.334 | 0.113 | 0.182 | 0.282 | 0.236 | 0.115 | 0.178 | 0.278 | 0.305 | 0.173 | 0.173 | 0.282 | 0.183 |
| 3-GS | 0.114 | 0.041 | 0.172 | 0.081 | 0.172 | 0.214 | 0.198 | 0.35 | 0.392 | 0 | 0.213 | 0.081 | 0.177 | 0.157 | 0.168 | 0.109 | 0.177 | 0.401 | 0.195 | **0.129** | 0.136 | 0.113 | 0.192 |
| 3-GO | 0.158 | 0.029 | 0.127 | 0.054 | 0.174 | 0.194 | 0.233 | 0.344 | 0.183 | 0.213 | 0 | 0.081 | 0.181 | 0.259 | 0.213 | 0.088 | 0.1 | 0.208 | 0.273 | 0.161 | 0.258 | 0.248 | 0.18 |
| 3-GH | 0.069 | 0.031 | 0.085 | 0.05 | 0.075 | 0.087 | 0.108 | 0.11 | 0.08 | 0.077 | 0.1 | 0 | 0.14 | 0.145 | 0.149 | 0.051 | 0.052 | 0.082 | 0.146 | 0.091 | 0.173 | 0.092 | 0.055 |
| 3-HH | 0.103 | 0.031 | 0.131 | 0.061 | 0.112 | 0.129 | 0.163 | 0.183 | 0.138 | 0.131 | 0.105 | 0.126 | 0 | 0.379 | 0.243 | 0.061 | 0.077 | 0.158 | 0.426 | 0.142 | 0.255 | 0.111 | 0.118 |
| 4-HO | 0.136 | 0.035 | 0.123 | 0.062 | 0.15 | 0.17 | 0.22 | 0.214 | 0.149 | 0.108 | 0.156 | 0.099 | 0.348 | 0 | 0.258 | 0.058 | 0.063 | 0.139 | 0.487 | 0.182 | 0.309 | 0.221 | 0.129 |
| 4-NO | 0.199 | 0.075 | 0.184 | 0.112 | 0.202 | 0.244 | 0.185 | 0.203 | 0.17 | 0.133 | 0.177 | 0.134 | 0.249 | 0.347 | 0 | 0.071 | 0.085 | 0.137 | 0.32 | 0.154 | 0.323 | 0.182 | 0.129 |
| K-SO | **0.131** | 0.052 | **0.185** | **0.105** | **0.19** | 0.214 | **0.18** | **0.237** | 0.22 | 0.201 | **0.207** | 0.066 | **0.145** | **0.167** | **0.161** | 0 | 0.481 | 0.177 | **0.169** | **0.109** | **0.176** | **0.162** | 0.184 |
| K-KR | **0.138** | 0.054 | 0.199 | 0.099 | 0.176 | 0.261 | 0.131 | 0.207 | 0.22 | 0.242 | 0.172 | 0.074 | **0.157** | **0.146** | 0.149 | 0.424 | 0 | 0.241 | 0.148 | **0.117** | 0.125 | 0.102 | 0.172 |
| G-SG | **0.136** | 0.053 | 0.139 | 0.085 | 0.25 | 0.236 | 0.269 | 0.319 | 0.247 | 0.394 | 0.188 | 0.065 | 0.16 | 0.175 | 0.147 | 0.111 | 0.204 | 0 | 0.226 | 0.101 | 0.135 | 0.192 | 0.147 |
| 4-SB | 0.169 | 0.057 | 0.194 | 0.099 | 0.283 | 0.226 | 0.37 | 0.354 | 0.173 | 0.149 | 0.24 | 0.121 | 0.429 | 0.685 | 0.36 | 0.088 | 0.101 | 0.222 | 0 | 0.127 | 0.334 | 0.209 | 0.147 |
| 5-BH | 0.072 | 0.033 | 0.094 | 0.045 | 0.065 | 0.09 | 0.095 | 0.087 | 0.104 | 0.057 | 0.102 | 0.063 | 0.118 | 0.163 | 0.119 | 0.037 | 0.044 | 0.069 | 0.136 | 0 | 0.223 | 0.14 | 0.066 |
| 5-DB | 0.176 | 0.056 | 0.177 | 0.095 | 0.158 | 0.184 | 0.185 | 0.182 | 0.134 | 0.106 | 0.181 | 0.099 | 0.318 | 0.553 | 0.323 | 0.07 | 0.091 | 0.138 | 0.39 | 0.256 | 0 | 0.168 | 0.133 |
| 5-BO | 0.109 | 0.055 | 0.118 | 0.077 | 0.183 | 0.165 | 0.23 | 0.201 | 0.15 | 0.095 | 0.142 | 0.083 | 0.118 | 0.206 | 0.16 | 0.05 | 0.063 | 0.103 | 0.209 | 0.093 | 0.183 | 0 | 0.103 |
| 6-NH | 0.16 | 0.08 | **0.226** | 0.115 | 0.292 | 0.252 | 0.243 | 0.327 | 0.201 | 0.139 | 0.239 | 0.091 | 0.208 | **0.313** | **0.298** | 0.089 | 0.117 | 0.153 | 0.277 | 0.127 | 0.215 | **0.245** | 0 |

**Table S9** Distribution of source meadows for 31 first-generation migrants based on filtered assignment probabilities in the genetic assignment test. Rows indicate sites where migrants were identified, and columns indicate putative source sites. For instance at site 1-BH, there is an identified immigrant coming with a high likelihood from site 6-NH.

The symbol # indicates the total number of migrants identified at a site and #a indicates the number of migrants that could actually be assigned to another sampling site. Red highlighting identifies sites were no migrants were found (rows) or to which no migrants were assigned to from other sites (columns). Note that the number of putative source sites is typically more than the number of assigned migrants. This is because up to three candidate source sites are shown if they had probabilities above the threshold of 0.1. The colour of the population labels corresponds to the cluster membership (Fig. 3 of the main text).

The actual probability that an individual belongs to the population from which it was sampled was calculated using a partially Bayesian criterion ([Rannala & Mountain 1997](#_ENREF_15)). We then compared the likelihood of exclusion to a distribution of likelihoods of 10,000 simulated genotypes in order to define a statistical threshold ([Paetkau *et al.* 2004](#_ENREF_14); [Underwood *et al.* 2007](#_ENREF_17)) with a type I error of 0.01. We excluded an individual from its sampling site when the probability for exclusion was above 95%. The identified migrants were then excluded from the the data set. The data-set without migrants served as the reference data to which migrants were assigned. Migrants were assigned to another sampled population when the probability was *P* ≥ 10% ([Underwood *et al.* 2007](#_ENREF_17)).

|  | # | #a | 1-BH | 1-DH | | 1-ST | G-BB | G-SG | K-KR | K-SO | 2-MV | 3-GS | 3-GO | 3-GH | 3-HH | 4-HO | 4-NO | 4-NO | 4-SB | 5-DB | 5-BH | 5-BO | 6-LM | 6-NH | 7-LS | 7-480 |
| --- | --- | --- | --- | --- | --- | --- | --- | --- | --- | --- | --- | --- | --- | --- | --- | --- | --- | --- | --- | --- | --- | --- | --- | --- | --- | --- |
|  |  |  | 0 | 0 | | 0 | 0 | 0 | 0 | 0 |  |  | 0 | 0 | 0 | 0 | 0 | 0 | 0 | 0 |  | 0 |  |  |  | 0 |
| 1-BH | 2 | 1 |  |  | |  |  |  |  |  |  |  |  |  |  |  |  |  |  |  |  |  |  | 0.170 |  |  |
| 1-DH | 3 | 1 |  |  | |  |  |  |  |  | 0.112 |  |  |  |  |  |  |  |  |  |  |  |  |  |  |  |
| 1-ST | 1 |  |  |  | |  |  |  |  |  |  |  |  |  |  |  |  |  |  |  |  |  |  |  |  |  |
| G-BB | 2 |  |  |  | |  |  |  |  |  |  |  |  |  |  |  |  |  |  |  |  |  |  |  |  |  |
| G-SG | 3 | 1 |  |  | |  |  |  |  |  |  | 0.201 |  |  |  |  |  |  |  |  |  |  |  |  |  |  |
| K-KR | 3 |  |  |  | |  |  |  |  |  |  |  |  |  |  |  |  |  |  |  |  |  |  |  |  |  |
| K-SO | 1 |  |  |  | |  |  |  |  |  |  |  |  |  |  |  |  |  |  |  |  |  |  |  |  |  |
| 2-MV | 2 |  |  |  | |  |  |  |  |  |  |  |  |  |  |  |  |  |  |  |  |  |  |  |  |  |
| 3-GS | 1 |  |  |  | |  |  |  |  |  |  |  |  |  |  |  |  |  |  |  |  |  |  |  |  |  |
| 3-GO | 0 |  |  |  | |  |  |  |  |  |  |  |  |  |  |  |  |  |  |  |  |  |  |  |  |  |
| 3-GH | 2 | 2 |  |  | |  |  |  |  |  | 0.475 |  |  |  |  |  |  |  |  |  |  |  | 0.702 |  | 0.261 |  |
| 3-HH | 1 |  |  |  | |  |  |  |  |  |  |  |  |  |  |  |  |  |  |  |  |  |  |  |  |  |
| 4-HO | 4 | 1 |  |  | |  |  |  |  |  | 0.160 |  |  |  |  |  |  |  |  |  | 0.114 |  |  |  |  |  |
| 4-NO | 1 |  |  |  | |  |  |  |  |  |  |  |  |  |  |  |  |  |  |  |  |  |  |  |  |  |
| 4-SB | 0 |  |  |  | |  |  |  |  |  |  |  |  |  |  |  |  |  |  |  |  |  |  |  |  |  |
| 5-DB | 0 |  |  |  | |  |  |  |  |  |  |  |  |  |  |  |  |  |  |  |  |  |  |  |  |  |
| 5-BH | 0 |  |  |  | |  |  |  |  |  |  |  |  |  |  |  |  |  |  |  |  |  |  |  |  |  |
| 5-BO | 0 |  |  |  | |  |  |  |  |  |  |  |  |  |  |  |  |  |  |  |  |  |  |  |  |  |
| 6-LM | 0 |  |  |  |  | |  |  |  |  |  |  |  |  |  |  |  |  |  |  |  |  |  |  |  |  |
| 6-NH | 1 |  |  |  | |  |  |  |  |  |  |  |  |  |  |  |  |  |  |  |  |  |  |  |  |  |
| 7-LS | 2 |  |  |  | |  |  |  |  |  |  |  |  |  |  |  |  |  |  |  |  |  |  |  |  |  |
| 7-480 | 0 |  |  |  | |  |  |  |  |  |  |  |  |  |  |  |  |  |  |  |  |  |  |  |  |  |

**Fig. S8** Geographic visualisation of asymmetric dispersal rates calculated with a) divMigrate online and b) GeneClass. Sites that are identified as sources are indicated by red dots, sites that are identified as sinks in blue. One site acts both as a source and a sink and is indicated with a purple dot. Geographic barriers as identified in Fig. 3 are shown with white dotted lines. For the DivMigrate analysis,only migration rates above a threshold of 0.2 and that show significant population differentiation are shown. The matrices on which the graphs are based are shown in Table S8 and S9.


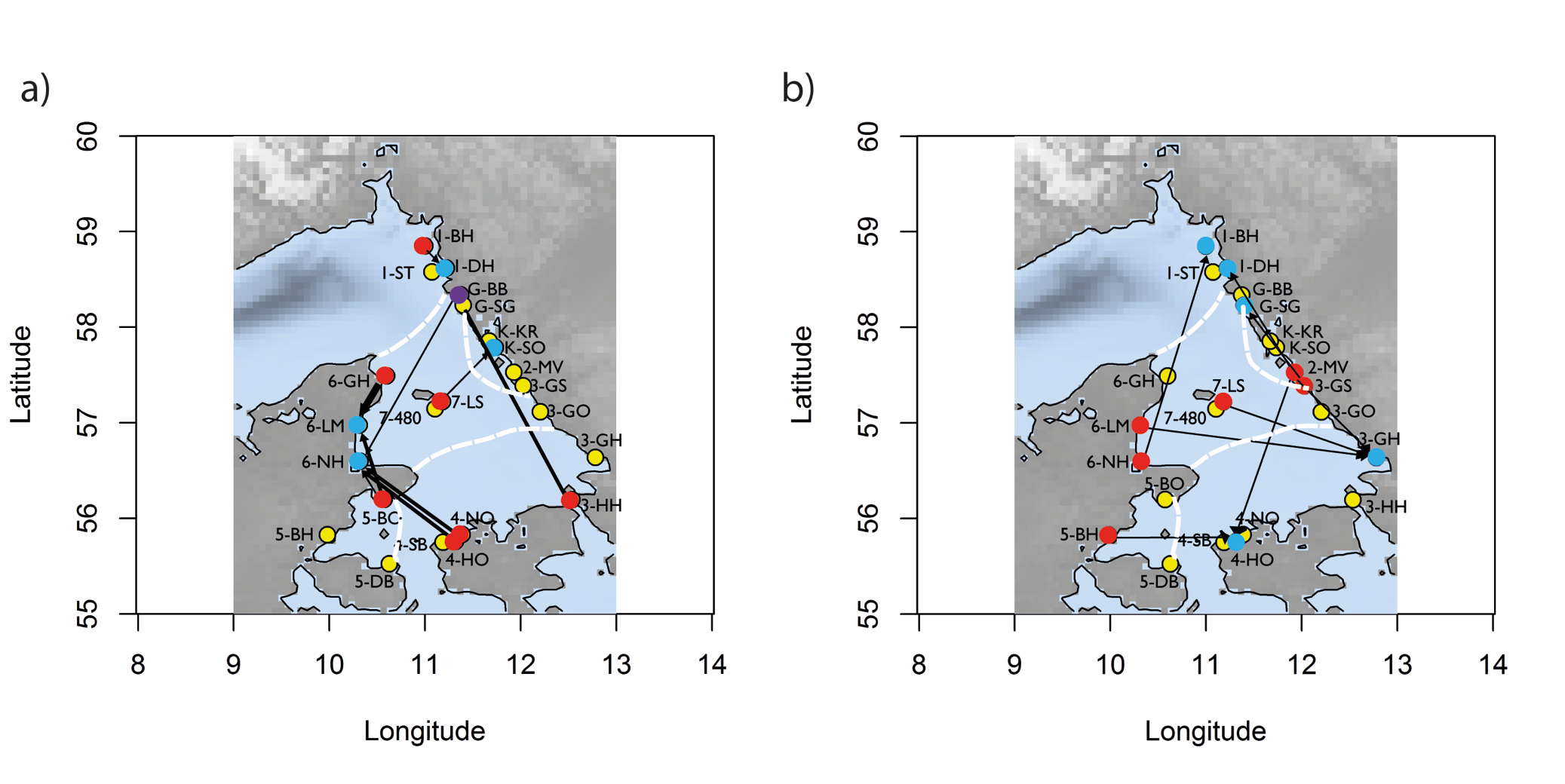


**Fig. S9** Heat map of **single generation** dispersal probability of *Zostera marina* based on the oceanographic particle model applied to the extant-mapped distribution. Sampling sites are ordered according to approximate geography in a clock-wise manner from the NE corner (Fig. 1 of main text). Dispersal is from column to row. For example, the dispersal probability from K-KR to K-SO is high (red), while it is low from K-KR to 1-ST (dark blue). Note that the highest dispersal probabilities are often within the population itself (e.g. 5-DB has the highest probability for self-retention, shown with a probability of 0.0396 for particle retention). Dark blue areas represent probabilities of zero. The oceanographic dispersal probability matrix on which the heat map is based can be found on dryad doixxx.


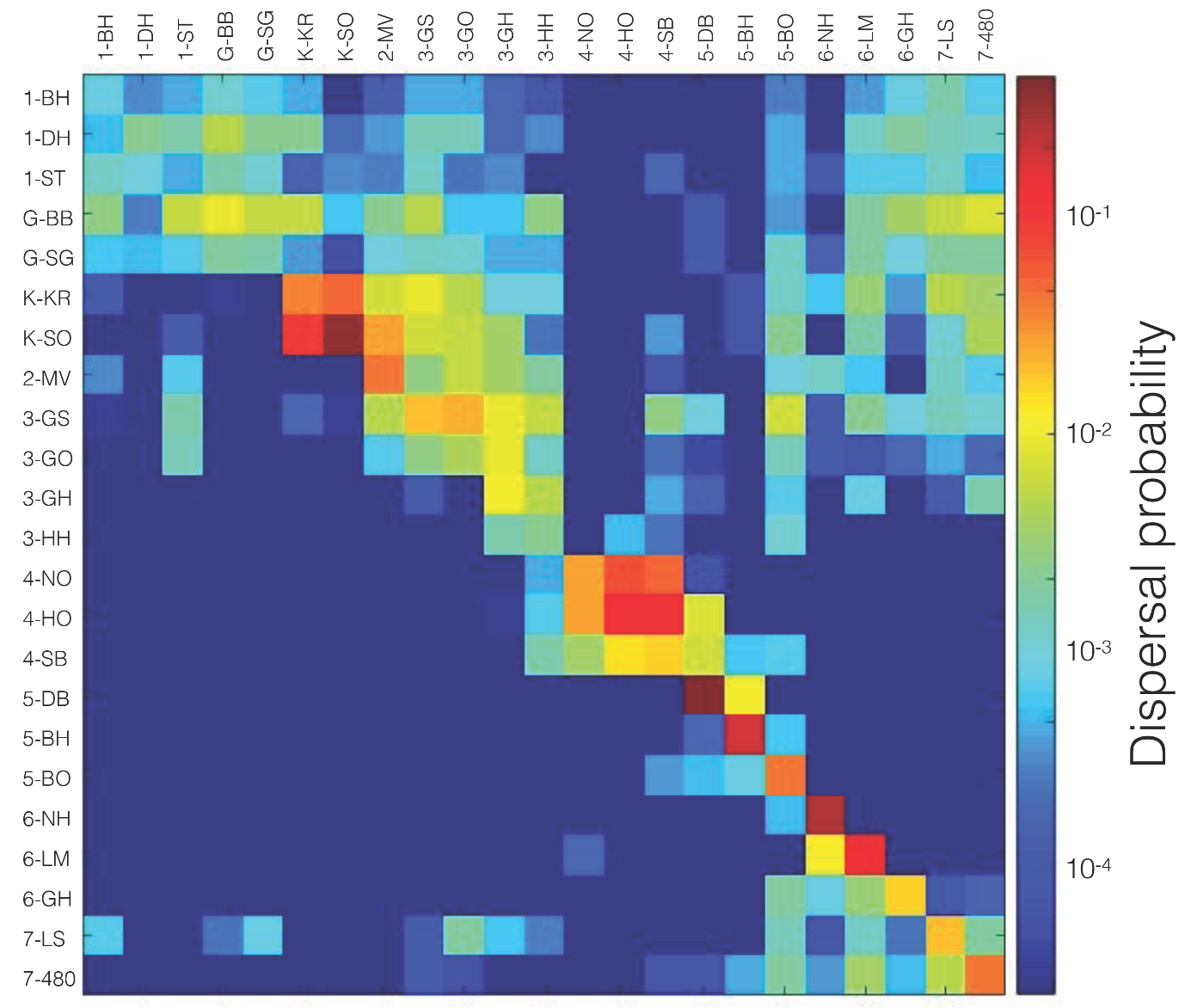


**Fig. S10** Heat map of **multi-generation** connectivity of *Zostera marina* based on the oceanographic particle model applied to the extant-mapped distribution of *Z. marina* (shown in Fig. 1 of the main text). Multi-generation dispersal, or connectivity, indicates the probability of stepping-stone dispersal across several generations (here 32 generations), and the connectivity between two sites includes all possible routes within the eelgrass habitat. Sampling sites are ordered according to approximate geography in a clock-wise manner from the NE corner (Fig. 1 of main text). Dispersal is from column to row. As an example, connectivity is high from 5-BH to 5-DB (red), but low from 5-BH to 1-ST (blue). Note that multi-generation connectivity may result in non-intuitive routes that is not possible during single-generation dispersal as shown in Fig. S9. Dark blue areas represent low probabilities (lowest probability: 8.89e-20). The oceanographic dispersal probability matrix on which the heat map is based can be found on dryad doixxx.


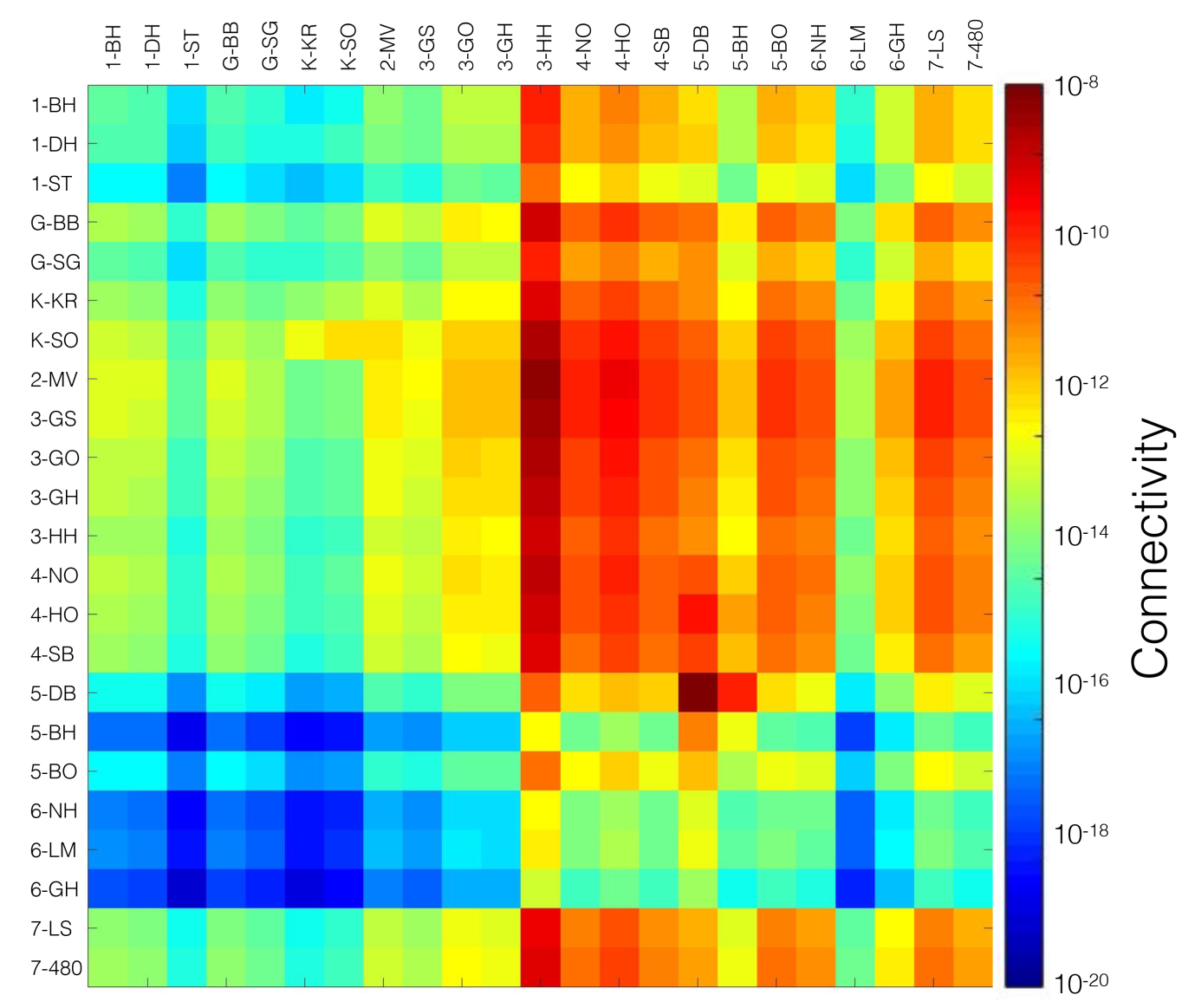


**Fig. S11** Heat map of multi-generation connectivity of *Zostera marina* based on the oceanographic particle model applied to the **mapped and inferred** **historic distribution** of *Z. marina* (shown in Fig. 1 of the main text). Multi-generation dispersal, or connectivity, indicates the probability of stepping-stone dispersal across several generations (here 32 generations), and the connectivity between two sites includes all possible routes within the eelgrass habitat. Sampling sites are ordered according to approximate geography in a clock-wise manner from the NE corner (Fig. 1 of main text). Dispersal is from column to row. As an example, connectivity is high from G-SG to 3-GS (orange), but low from G-SG to 5-BH (blue). Note that multi-generation connectivity may result in non-intuitive routes that is not possible during single-generation dispersal as shown in Fig. S9. Dark blue areas represent dispersal probabilities as low as 2.23e-18. The oceanographic dispersal probability matrix on which the heat map is based can be found on dryad doixxx.

**
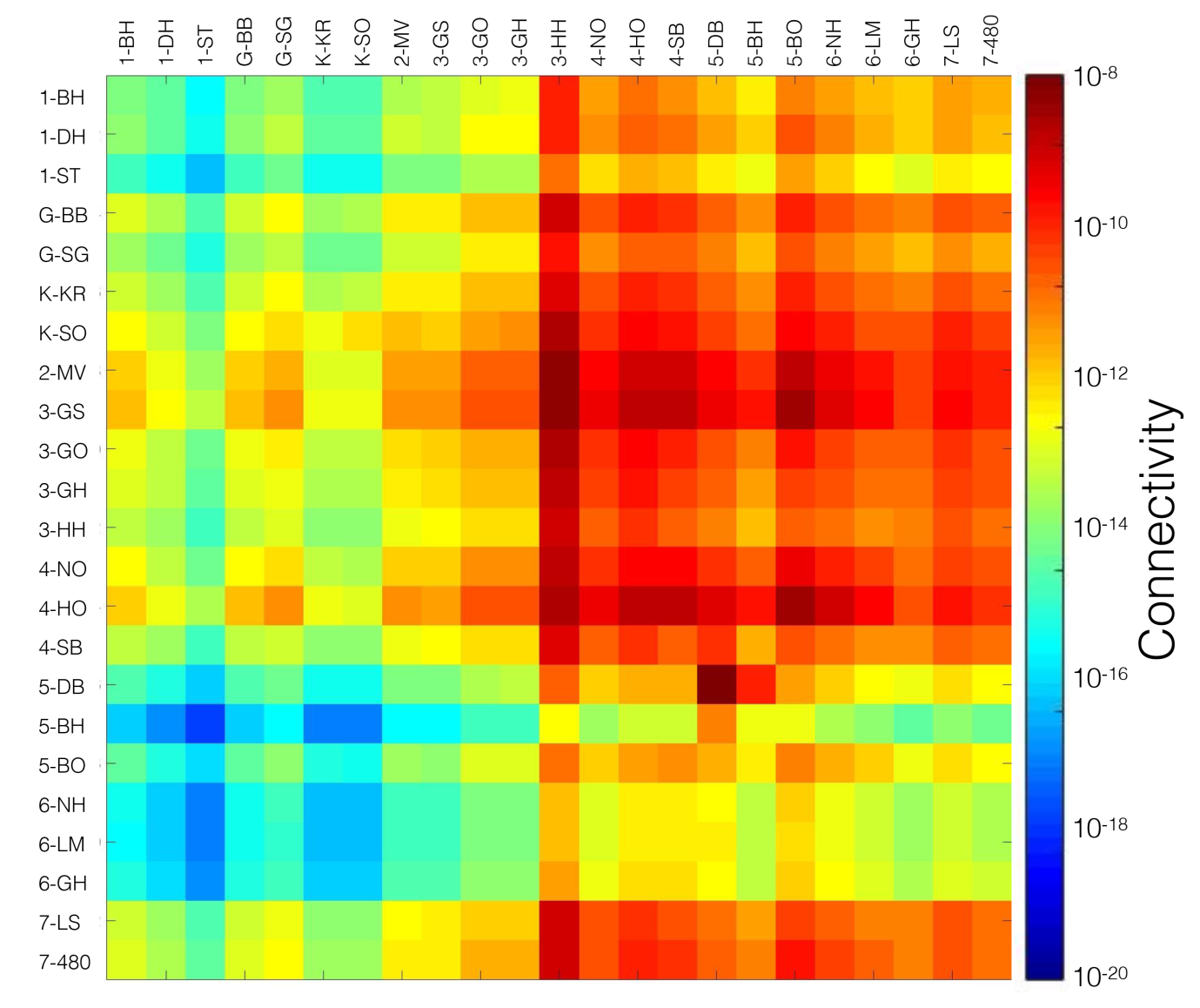
**

**Fig. S12** Heat map of the change in multi-generation connectivity of *Zostera marina* calculated as the ratio between connectivities based on inferred historic distribution (Fig. S11) and on the currently mapped distribution (Fig. S10) of *Z. marina*. Connectivity was estimated from the oceanographic particle model. Sampling sites are ordered according to approximate geography in a clock-wise manner from the NE corner (Fig. 1 of main text). Change in connectivity is read from column to row. As an example, connectivity is expected to have been considerably higher for the inferred historic distribution for site 6-LM acting as sources, and for 6-GH acting as a sink.


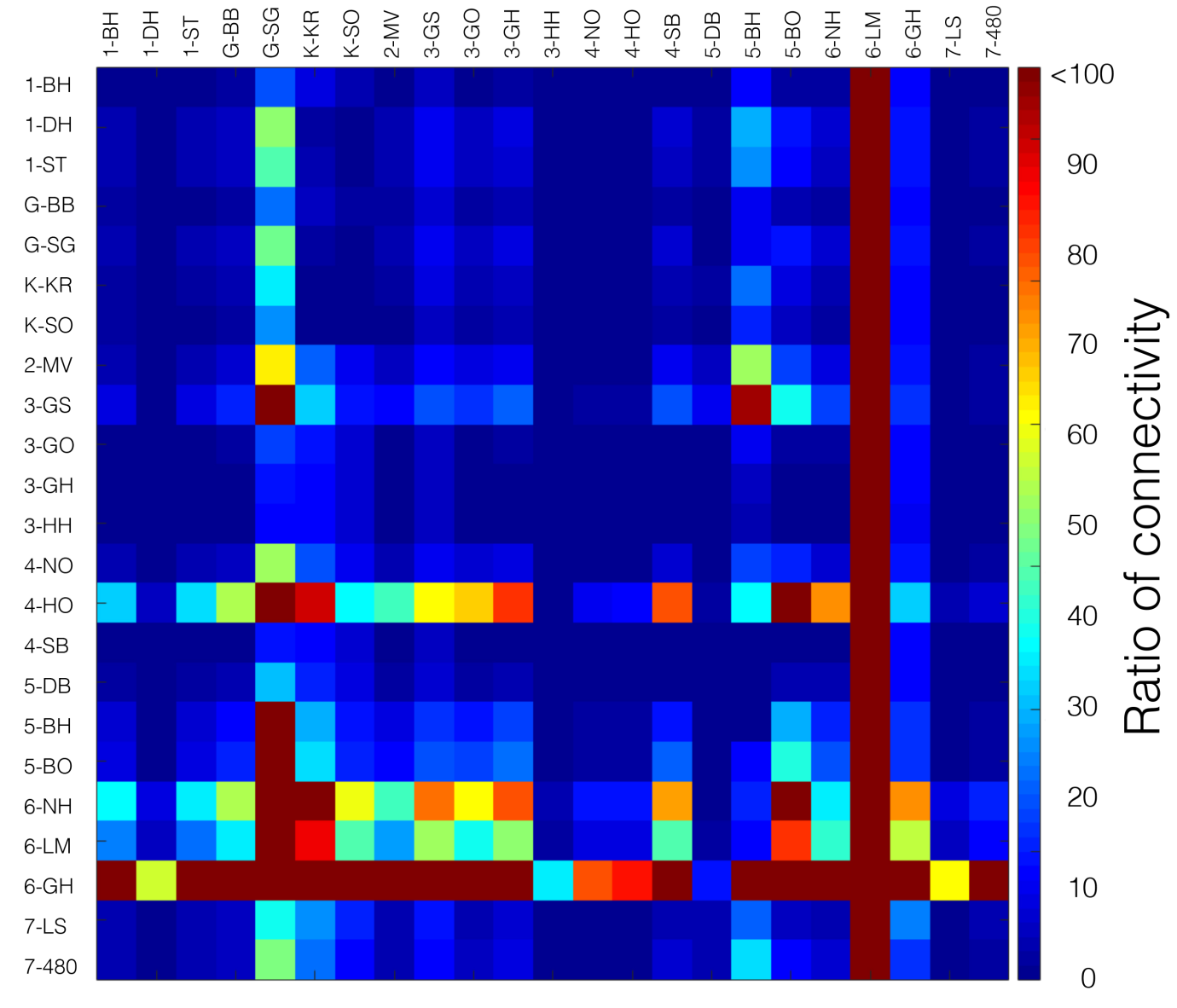


**References**

Antao T, Lopes A, Lopes R, Beja-Pereira A, Luikart G (2008) LOSITAN: a workbench to detect molecular adaptation based on a Fst-outlier method. *BMC bioinformatics* **9**, 323.

Earl DA, von Holdt BM (2012) STRUCTURE HARVESTER: a website and program for visualizing STRUCTURE output and implementing the Evanno method. *Conservation Genetics Resources* **4**, 359-361.

Evanno G, Regnaut S, Goudet J (2005) Detecting the number of clusters of individuals using the software structure: a simulation study. *Molecular Ecology* **14**, 2611-2620.

Excoffier L, Lischer HEL (2010) Arlequin suite ver 3.5: a new series of programs to perform population genetics analyses under Linux and Windows. *Molecular Ecology Resources* **10**, 564-567.

Falush D, Stephens M, Pritchard JK (2003) Inference of population structure using multilocus genotype data: linked loci and correlated allele frequencies. *Genetics* **164**, 1567-1587.

Foll M, Gaggiotti O (2008) A genome-scan method to identify selected loci appropriate for both dominant and codominant markers: a Bayesian perspective. *Genetics* **180**, 977-993.

Hedrick PW (2005) A standardized genetic differentiation measure. *Evolution* **59**, 1633-1638.

Jost LOU (2008) GST and its relatives do not measure differentiation. *Molecular Ecology* **17**, 4015-4026.

Keenan K, McGinnity P, Cross TF, Crozier WW, Prodöhl PA (2013) diveRsity: An R package for the estimation and exploration of population genetics parameters and their associated errors. *Methods in Ecology and Evolution* **4**, 782-788.

Keil K (2011) *Adaptation to contrasting habitats and heterozygosity-fitness correlations in eelgrass (Zostera marina)*, Christians-Albrechts-Universität, Kiel, Germany.

Kopelman NM, Mayzel J, Jakobsson M, Rosenberg NA, Mayrose I (2015) Clumpak: a program for identifying clustering modes and packaging population structure inferences across K. *Molecular Ecology Resources* **15**, 1179-1191.

Olsen JL, Coyer JA, Chesney B (2014) Numerous mitigation transplants of the eelgrass *Zostera marina* in southern California shuffle genetic diversity and may promote hybridization with *Zostera pacifica*. *Biological Conservation* **176**, 133-143.

Olsen JL, Coyer JA, Stam WT, Moy FE, Christie H, Jørgensen NM (2013) Eelgrass *Zostera marina* populations in northern Norwegian fjords are genetically isolated and diverse. *Marine Ecology Progress Series* **486**, 121-132.

Paetkau D, Slade R, Burden M, Estoup A (2004) Genetic assignment methods for the direct, real-time estimation of migration rate: a simulation-based exploration of accuracy and power. *Molecular Ecology* **13**, 55-65.

Rannala B, Mountain JL (1997) Detecting immigration by using multilocus genotypes. *Proceedings of the National Academy of Sciences* **94**, 9197-9201.

Sundqvist L, Keenan K, Zackrisson M, Prodohl P, Kleinhans D (2016) Directional genetic differentiation and relative migration. *Ecology and Evolution* **6**, 3461-3475.

Underwood J, Smith L, Van Oppen M, Gilmour J (2007) Multiple scales of genetic connectivity in a brooding coral on isolated reefs following catastrophic bleaching. *Molecular Ecology* **16**, 771-784.
